# Unraveling the Kinetics of the 10–23 RNA-Cleaving DNAzyme

**DOI:** 10.3390/ijms241813686

**Published:** 2023-09-05

**Authors:** Aida Montserrat Pagès, Maarten Hertog, Bart Nicolaï, Dragana Spasic, Jeroen Lammertyn

**Affiliations:** 1 Department of Biosystems, Biosensors Group, KU Leuven—University of Leuven, 3001 Leuven, Belgium; 2 Department of Biosystems, Postharvest Group, KU Leuven—University of Leuven, 3001 Leuven, Belgium

**Keywords:** RNA-cleaving DNAzymes, reaction kinetics, mathematical modeling

## Abstract

DNA-based enzymes, or DNAzymes, are single-stranded DNA sequences with the ability to catalyze various chemical reactions, including the cleavage of the bond between two RNA nucleotides. Lately, an increasing interest has been observed in these RNA-cleaving DNAzymes in the biosensing and therapeutic fields for signal generation and the modulation of gene expression, respectively. Additionally, multiple efforts have been made to study the effects of the reaction environment and the sequence of the catalytic core on the conversion of the substrate into product. However, most of these studies have only reported alterations of the general reaction course, but only a few have focused on how each individual reaction step is affected. In this work, we present for the first time a mathematical model that describes and predicts the reaction of the 10–23 RNA-cleaving DNAzyme. Furthermore, the model has been employed to study the effect of temperature, magnesium cations and shorter substrate-binding arms of the DNAzyme on the different kinetic rate constants, broadening the range of conditions in which the model can be exploited. In conclusion, this work depicts the prospects of such mathematical models to study and anticipate the course of a reaction given a particular environment.

## 1. Introduction

Enzymes are naturally occurring molecules exhibiting a catalytic functionality, which play a crucial role in biological and industrial processes, placing them at the heart of enzyme-based biosensors [1]. For many years, the scientific dogma that only proteins could have enzymatic activity was widely accepted. However, in 1982, Cech et al. showed evidence of a self-splitting RNA, named ribozyme, causing a transformation in the field [2]. A decade later, the work from Santoro and Joyce revolutionized the field again by synthesizing the first DNA-based enzyme [3]. DNA enzymes, also known as DNAzymes, are DNA sequences able to catalyze chemical reactions in a similar manner as protein enzymes. However, contrary to ribozymes or protein enzymes, DNAzymes cannot be found in nature and are generated through an in vitro selection process [4]. Since their discovery, many DNAzymes have been selected, exhibiting a wide variety of functionalities, such as redox [5], ligation [6], phosphorylation [7] or RNA cleavage [8].

In the biosensing field, RNA-cleaving DNAzymes have attracted significant attention [9] as signal generation elements due to their numerous advantages compared to protein or RNA analogues [10]. Some of these advantages include low manufacturing cost, their stability over a wide condition range and their ability to work at different temperatures [11,12]. They have been used for the detection of metal ions in different samples [13], nucleic acid detection [14,15] and proteins, the last when combined with aptamers [16,17]. Furthermore, their high specificity makes DNAzymes an excellent choice for multiplex target detection [18,19]. Another important field of application is therapeutics, in which RNA-cleaving DNAzymes are a promising tool to modulate the expression of certain genes [20,21]. Nevertheless, in this field, some challenges still need to be overcome, the main one being the fact that the low concentrations of cofactors present inside cells hinder the performance of DNAzymes [22,23].

Among the several selected RNA-cleaving DNAzymes, the 10–23 is the most extensively studied [8]. It has a catalytic loop of 15 nucleotides, flanked on both sides by the substrate-binding arms, and catalyzes the cleavage of RNA sequences and DNA/RNA hybrids [24]. For many years, researchers have studied the reaction and the structure–function relation [25,26,27], but due to a lack of sufficient structural information, its reaction mechanism has not been unraveled until recently [28]. Furthermore, relevant information about the performance of this DNAzyme has been obtained over the years, such as the dependence on a divalent cation (i.e., magnesium, Mg^2+^), pH and temperature [11]. Many studies have focused on the functionality and role of each core nucleotide [29,30], and even their different functional groups [31]. Additionally, some studies have identified relations between the hybridization free energy of the substrate-binding arms and the rate of the reaction [32]. However, most of these studies are based on the overall enhancement or reduction of signal generation to describe the kinetics of the reaction, and, to the best of our knowledge, none of the available studies have investigated how the reaction conditions influence each reaction step individually. Having insight on how the different reaction steps are affected by the reaction conditions would allow for a better and more precise control of the reaction, as changing the conditions (e.g., increasing the temperature or decreasing the concentration of the cofactor) might lead to opposite effects on different reaction steps, resulting in an undesired output. Besides the reaction conditions, the relation between the length of the substrate-binding arms and the reaction temperature is a relevant aspect to study, especially in the biosensing field, since there are some applications that work at room temperature [16,33,34]. Additionally, the possibility to observe which reaction step is affected provides a deeper understanding of the reaction mechanism.

**Figure 1:**
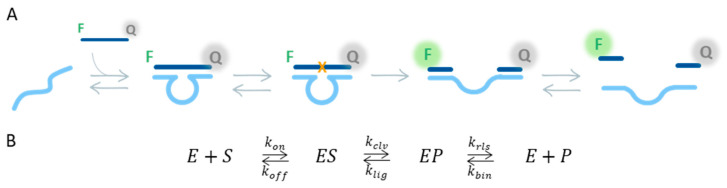
Overview of the procedure for development and validation of the mathematical model. (**A**,**B**) Proposed reaction mechanism of the RNA-cleaving 10–23 DNAzyme, illustrated schematically and by the minimal kinetic scheme, respectively. DNAzymes (E) contain two substrate-binding arms, adjacent to the catalytic core, which bind the substrate (S). The hybridization of E and S forms a complex (ES), which cleaves the phosphodiester bond between the RNA bases positioned in the middle of the substrate sequence (depicted in A with an orange cross), resulting in EP. After cleavage, the catalytic core stretches, increasing the end-to-end distance. Subsequently, the product sequences are released, and E is able to initiate a new reaction. In this kinetic scheme, P refers to the product strand that dissociates most slowly. The reaction is monitored by labeling the ends of the S with a fluorophore (F) and a quencher (Q). Upon cleavage of the S sequence, the distance between the F and Q increases, resulting in a fluorescent signal increase. The association and dissociation of E and S are indicated by *k_on_* and *k_off_*. The cleavage and ligation rates are defined by *k_clv_* and *k_lig_*, and the rates for release and binding of P are represented by *k_rls_* and *k_bin_*.

Therefore, in this work, we present for the first time a mathematical model to describe the cleavage reaction catalyzed by the 10–23 DNAzyme. The reaction mechanism employed has been defined based on the available studies and the similarities between RNA-cleaving DNAzymes and hammerhead ribozymes (Figure 1A,B) [11,26,28,35,36]. Through the fitting of experimental curves for different substrate concentrations and the estimation of the rate constants, the proposed reaction mechanism is validated. Finally, we expand the model by implementing the dependence of the reaction kinetics on the temperature and the concentration of the reaction cofactor Mg^2+^, and estimating the rate constants for a DNAzyme with shorter substrate-binding arms that cleaves its substrate at room temperature.

To accomplish this, we measure the product generation over time by labeling the substrate sequence (i.e., single-stranded DNA sequence with two RNA bases on the cleavage site) labeled with a fluorophore (F) and a quencher (Q) on both ends. Upon cleavage, the distance between them increases, resulting in an increase in the fluorescent signal (Figure 1A) [37]. The kinetic curves are monitored for several concentrations of substrate, while keeping a fixed concentration of DNAzyme. By means of ordinary differential equations (ODEs) describing the reaction in Figure 1B, we estimate the rate constants using an enhanced scatter search (ESS) and the least-squares nonlinear optimization (lsqnonlin) method. Next, the 95% confidence interval (CI) of these parameters is calculated from the distribution after bootstrap resampling. Lastly, Monte Carlo simulations are carried out to assess the output based on the variability of the input variables. Following the development of the model, we demonstrate the feasibility of predicting unknown conditions by simulating the model output of a specific condition, not included during the optimization process, and comparing it with the experimental data obtained under the same conditions. Moreover, we study the robustness of the model by executing a sensitivity analysis and identify the rate-limiting steps of the reaction. Lastly, we demonstrate the usefulness of the model by fitting curves measured under several temperatures and concentrations of Mg^2+^ next to those measured for DNAzyme with shorter substrate-binding arms to investigate the dependence of the different reaction steps on these conditions. As such, we describe the first mathematical model to extract the rate constants of each individual reaction step from the fluorescent signal generated over time. Additionally, the model can be used to predict the velocity of the reaction for specific experimental conditions. Understanding the role of the different steps in the overall reaction is essential to select which steps should be modified in order to further optimize or tailor the speed of the reaction to specific needs.

## 2. Results and Discussion

### 2.1. Activity Measurements

**Figure 2:**
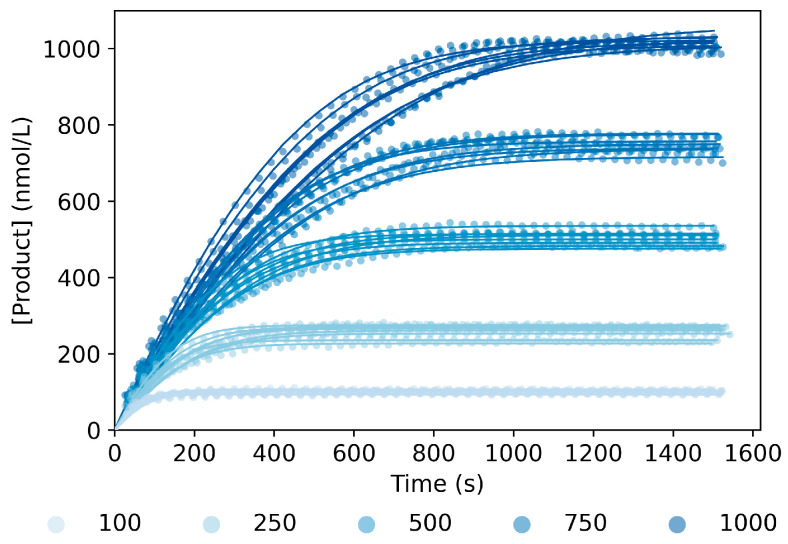
Product generation measured experimentally (symbols) and fitted curves (solid lines) for several concentrations of Substrate_Lg_ (100–1000 nmol/L), 10 nmol/L of DNAzyme_Lg_ and 20 mmol/L of Mg^2+^ at 55 °C. For each substrate concentration, there were eight independent repetitions. All experimental curves were fitted simultaneously, using the No Ligation model described in Figure S2B. The parameter estimated can be found in Table S4.

To study the kinetics of the 10–23 DNAzyme catalyzed reaction, we monitored the conversion of a DNA–RNA hybrid substrate into product by fluorescently labeling the substrate as depicted in Figure 1A. The reaction was studied for 10 nmol/L of the DNAzyme with long substrate-binding arms (DNAzyme_Lg_) and several concentrations of the corresponding long substrate (Substrate_Lg_, 100–1000 nmol/L), and the measurements were calculated at the suggested working temperature of 55 °C [38] and 20 mmol/L of Mg^2+^ (Figure 2, symbols). In order to determine whether all substrate sequences had been converted into product, we compared the fluorescence intensity of the reaction curves with the intensity of a sequence representing half of the original substrate (i.e., one of the generated products). The result of such comparison confirmed full completion of the reaction (Figure S1).

### 2.2. Model Optimization

To model the DNAzyme reaction, we divided the reaction into several steps following the mechanism of the reaction instead of using a general parameter to describe the whole reaction course (*k_obs_*). In this manner, we obtained kinetic information for each reaction step and a more detailed characterization of the DNAzyme activity. The reaction mechanism employed here was based on the similarities with the hammerhead ribozyme, the initial work performed by Santoro and Joyce and the recent findings from Borggräfe et al., shown in Figure 1B. The reaction starts with the hybridization of the substrate and the DNAzyme, thereby forming the DNAzyme–Substrate complex (ES). This is followed by the cleavage of the phosphodiester bond between the RNA bases and stretching of the catalytic core, resulting in the DNAzyme–Product complex (EP). Although the study by Borggräfe et al. proposes several intermediate steps between the ES and EP complexes, we opted for a reduced version, in which these intermediate steps are described by *k_clv_*. Their mechanism includes the stabilization and activation of the ES complex via ion binding, resulting in a too complex model for the experimental data we had available. Finally, the two reaction products dissociate from the DNAzyme, allowing the latter to start a new cycle. Although the reaction generates two products, for simplicity, the last step of the reaction (i.e., the release of the product strands) refers only to the slowest one, which, according to the Gibbs free energy, happens at the 5′ side of the substrate sequence (P). As mentioned earlier, upon cleavage of the substrate sequence, the DNAzyme catalytic core changes the conformation [28,36], increasing the distance between the F and Q. Therefore, assuming that both EP and P contribute equally to the final measured fluorescence (Figure 1A), we constructed the model considering the sum of the EP and P concentrations to be compared with the experimental data.

Considering the aspects mentioned in the previous paragraph and using the law of mass action, we wrote the ODEs (Equations (1)–(5)) describing the reaction mechanism, where each step was formalized by the forward and the backward reaction, and the corresponding rate constant (Figure 1B). To start with, we assumed equilibrium reactions for all the steps. To optimize the parameter estimates for the full-equilibrium model version, we followed the steps described in the corresponding section of Materials and Methods. From the estimates obtained (Full Equilibrium column in Table S3), we could observe that the rate constant of the ligation reaction (*k_lig_*) was hundred times smaller than the forward reaction, i.e., the cleavage of the substrate (*k_clv_*). These results suggested that the reaction had a stronger preference for the cleavage of the substrate than the ligation of the product strands, which was consistent with the observations from Santoro and Joyce [11]. Additionally, the estimated *k_lig_* was not significantly different from zero due to the large standard deviation. Therefore, we excluded this reaction step and refitted the model (No Ligation, Figure S2A). To compare the performance between model versions, we used the estimates and standard deviation of the parameters and Akaike’s information criterion (AIC), which is a measure to evaluate the goodness of fit, including a penalty for the number of parameters to estimate [39]. As can be seen from the obtained results (No Ligation column in Table S3), excluding *k_lig_* from the model resulted in a slightly larger AIC, but all the parameters estimates were significant since the standard deviation of the estimates was lower. Similarly, we also observed that in both models, the release rate constant (*k_rls_*) was a hundred times larger than the one referring to the binding of the product and the DNAzyme (*k_bin_*). Thus, we estimated the rate constants, excluding *k_bin_*, as well (No Binding, Figure S2B), which caused an increase in the AIC and larger standard deviations and non-significant estimates (No Binding column in Table S3). A possible explanation for this decrease in the performance could be that, by removing two reaction parameters, we were excessively simplifying the model, and, as such, it was not able to provide a good fit. Based on the results obtained, we could infer that only removing the ligation step led to the best fit of the experimental data, while maintaining a significant estimation of the rate constants. Additionally, this model version was in agreement with the previous findings [11]. Therefore, we selected the No Ligation version of the model for subsequent fittings. To account for pipetting inaccuracies and variability during the manual preparation of the reaction solutions, we introduced two terms describing the relative error between the target and the actual concentration of DNAzyme and substrate, named ${Err}_{E}$ and ${Err}_{S}$. We estimated the value of these errors for each reaction curve independently and used them to initiate the state variables using Equations (6) and (7), resulting in a model able to follow each reaction curve with good accuracy (Figure 2, solid lines).

The CI of the parameter estimates obtained during the calibration of the model using the lsqnonlin were calculated under the assumption of normality, which, in the case of nonlinear models, is often not true. Therefore, to obtain a more accurate CI, we ran the bootstrapping feature of the software, which applies an error-based resampling method to resample the original data, generating random look-a-like data sets, which were subsequently used for model fitting [40]. During this step, we generated one hundred bootstrap data sets, resulting in one hundred sets of parameter estimates, from which we calculated their 95% CI (Table 1), thus accounting for any non-normality in the parameters’ distributions (Table S4), and their possible correlation (Figure S3). Additionally, this step provided information about the variation of the reaction input. Regarding the ${Err}_{S}$, as depicted in Figure S4A, the values were between 0.89 and 1.12, indicating less than a 12% error in terms of the substrate. Similarly, focusing on ${Err}_{E}$ (Figure S4B), the values observed were slightly larger than for ${Err}_{S}$, being between 0.74 and 5.38. Nevertheless, we could observe that these larger relative errors were only obtained for the lowest concentration of substrate (100 nmol/L), while for the upper value, hardly any large relative error was present. A possible explanation could be that due to the short duration of the reaction, the information content was too low for a more accurate estimation.

**Figure 3:**
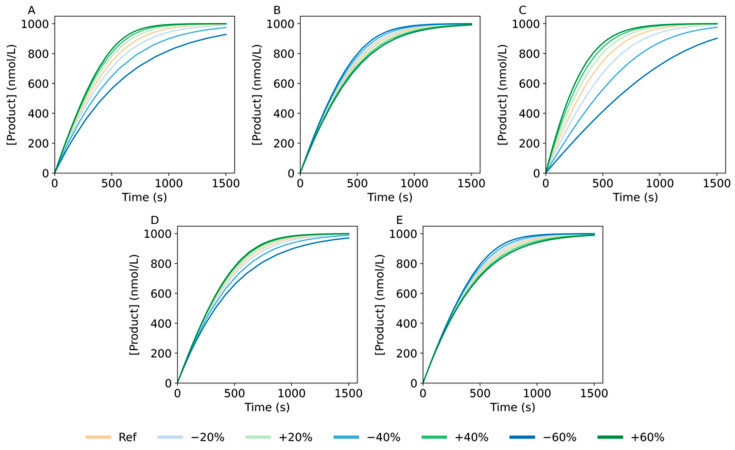
Evaluation of the model output based on the alteration of the kinetic rate constants for a Substrate_Lg_ concentration of 1000 nmol/L. To perform this analysis, the rate constants were systematically evaluated in a range of ±60%, based on the estimate values shown in Table S4. The process was performed for each of the rate constants independently: (**A**) *k_on_*, (**B**) *k_off_*, (**C**) *k_clv_*, (**D**) *k_rls_* and (**E**) *k_bin_*.

To further understand the significance of each reaction step in the global product generation, we performed a sensitivity analysis where we systematically increased or decreased one of the rate constants and simulated the output of the reaction. Although the analysis was performed for all the substrate concentrations included in the training data set, the most significant changes were observed for the highest substrate concentration, 1000 nmol/L (Figure 3). From the results obtained, we could see that *k_off_* and *k_bin_* are the most robust parameters, as not much change in the output was observed when applying up to a 60% change in these rate constants. Considering these two parameters refer to backward steps, it is interesting to see that even if their value varies up to 60%, the outcome of the reaction is not much affected. Thus, the reaction conditions could be modified to favor the forward steps, without significant impact on the backward steps. On the other hand, the most sensitive parameter was *k_clv_*, followed by *k_on_*, as a variation of their value resulted in a large alteration of the reaction output. Interestingly, we noticed a different change of the global reaction rate, depending on whether the rate constant was increased or decreased. A possible explanation for this behavior would be that when increasing a rate constant, the overall increase might be limited by another rate constant, and, as such, accelerating that step would not lead to an additional increase in the total reaction rate. From the biological point of view, the upper limit of *k_clv_* could be explained by the maximal speed achievable with the chemical groups available in the nucleotides of the core sequence, as shown by Breaker et al. [41]. Based on the results obtained, the DNAzyme catalyzed reaction was most likely limited by the speed of the cleavage, closely followed by the hybridization step. The findings from this sensitivity analysis are in accordance with the results from Borggräfe et al. since the step they determined as potentially rate limiting is part of the step captured by *k_clv_* [28]. Notwithstanding, in multiple turnover conditions (i.e., excess of substrate), in order to substantially increase the overall rate of the DNAzyme reaction, multiple reaction steps should be accelerated. Modifying the experimental conditions would have an impact on several reaction steps, thus being a prospective approach to modulate the velocity of the reaction.

### 2.3. Model Validation

**Figure 4:**
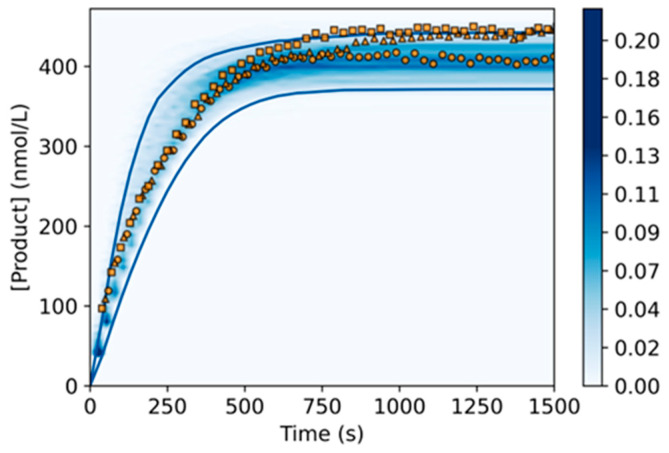
Validation of the model by predicting the time course of the DNAzyme_Lg_ reaction for a test concentration of 400 nmol/L of Substrate_Lg_ and 10 nmol/L of DNAzyme_Lg_. The colormap represents the frequency of the distributions of the modeled product as a function of time; for each time point, the sum of all the frequencies equals to 1. The three orange symbols indicate the three validation experiments, and the blue solid lines depict the 95% CI. The reaction was performed at 55 °C and in the presence of 20 mmol/L of Mg^2+^.

With the aim of evaluating the predictive value of the model, we predicted the response of the reaction for four concentrations not included in the training set: one within the concentration range studied and three outside. To achieve that, we first simulated the product generation for 400 nmol/L of Substrate_Lg_ and 10 nmol/L of DNAzyme_Lg_ using Monte Carlo simulations, a technique to simulate the probability of the model outcome based on the uncertainty of the model parameter values [42]. In this case, we simulated the product increase over time based on the possible variability introduced by the distribution of *Err_S_* and *Err_E_*. Afterwards, we compared the corresponding experimental curves with the probability of the simulated reaction output. As can be observed in Figure 4, we obtained a good agreement between the model predictions and the experimental data, demonstrating in this manner the possibility to predict the time course of the DNAzyme_Lg_ reaction given a specific set of reaction conditions. In a next step, to stretch the limits of the model, we simulated the reaction kinetics for three additional Substrate_Lg_ concentrations that fell outside the range used in the fitting process (i.e., 50, 1250 and 1500 nmol/L). From the comparison with the experimental data, we could observe that for smaller concentrations (Figure S5A), the model was able to predict reasonably well the course of the reaction. Nevertheless, for higher concentrations (Figure S5B,C), discrepancies were noticed, as the model predicted a faster reaction than what was observed in the experimental data. Based on the results obtained, we could conclude that the model is able to appropriately anticipate the product generation up to a concentration of 1000 nmol/L of Substrate_Lg_. Since the differences observed increase with larger concentrations of substrate, we hypothesized that these results are due to a saturation of the DNAzymes by the substrate available in the solution, which was not covered by the current model approach. Thus, from the signal amplification point of view, large ratios between the DNAzyme and the substrate could decrease the speed at which the signal is being generated, resulting in a lower performance of the bioassay.

### 2.4. Temperature Dependence

**Figure 5:**
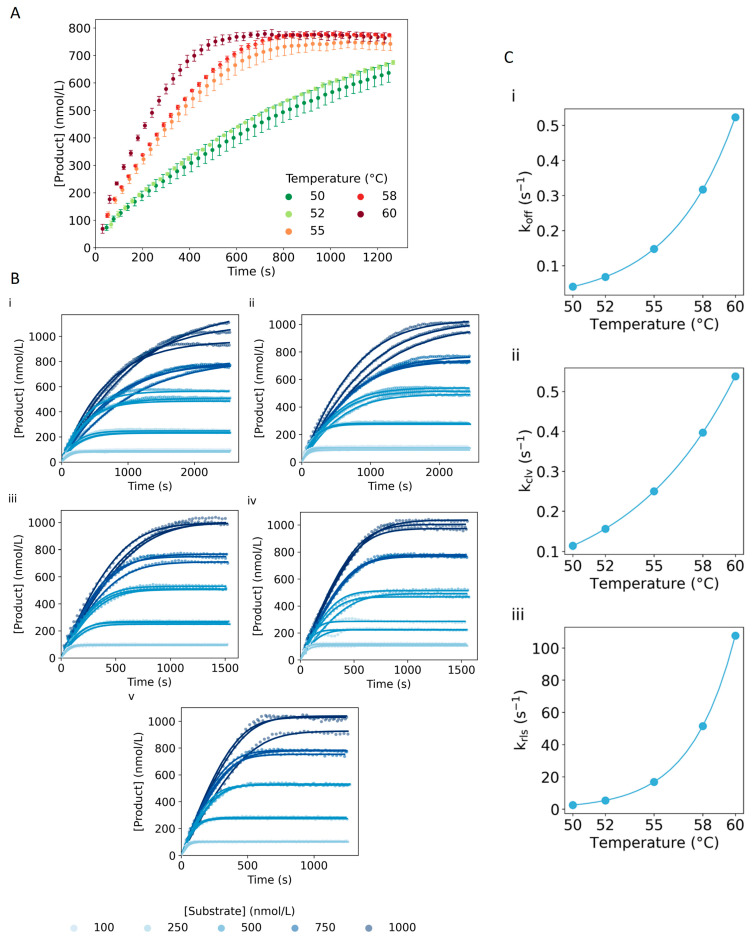
Temperature dependence of the reaction. (**A**) Generation of product over time for 10 nmol/L DNAzyme_Lg_ and 750 nmol/L of Substrate_Lg_ measured at several temperatures (50–60 °C) and 20 mmol/L of Mg^2+^. The error bars represent the standard deviation from three independent repetitions. (**B**) Comparison of the product increase between the experimental curves (circles) and the model (solid lines) for different temperatures: (i) 50 °C, (ii) 52 °C, (iii) 55 °C, (iv) 58 °C and (v) 60 °C. The reaction was measured for 10 nmol/L of DNAzyme_Lg_ and several Substrate_Lg_ concentrations (100–1000 nmol/L) in the presence of 20 mmol/L of Mg^2+^. The parameters estimated can be found in Table S6. (**C**) Scatter plot depicting the relationship between (i) *k_off_*, (ii) *k_clv_* and (iii) *k_rls_* with the temperature.

Temperature is an important factor in the kinetics of any enzymatic reaction [43]. Thus, we extended the model to study such dependency by monitoring the DNAzyme reaction at four additional temperatures (50, 52, 58 and 60 °C), which were below the melting temperature (Tm) of the DNAzyme–Substrate complex (67 °C) and above the Tm of the DNAzyme–Product complex (42.4 and 40.6 °C for the 5′ and the 3′ side, respectively). Overall, we observed an increase in the reaction speed when increasing the temperature (Figure 5A). Additionally, we modified the ODEs to include the temperature dependence on each rate constant using the relation defined by Arrhenius (Equation (8)) and estimated the *E_a_* for each reaction step following the process described in the Materials and Methods section. DNA hybridization studies have determined that the association of two DNA strands has a high temperature dependence and follows an anti-Arrhenius behavior, meaning that the rate of the reaction diminishes when increasing the temperature [44,45,46]. This behavior is associated with the instability increase in initial contacts between two DNA strands. Hence, in accordance with these findings, we performed the estimation of the *E_a_* starting with much lower initial values for the association and product binding steps.

From the estimated values (column named All E_a_ in Table S5), we could observe a larger *E_a_* value for the substrate dissociation step than for the association, indicating that the formation of the ES complex would be more impacted by the larger dissociation rates when the temperature increases. We observed a similar effect for the *E_a_* values referring to the release and binding of the product. Consequently, since *k_on_* and *k_bin_* showed minimal temperature dependence, we evaluated the ability of the model to fit the data when removing either one parameter or both, reducing in this manner the complexity of the model. The comparison of the different model variations was performed using the estimates and standard deviations of *E_a_*, and the AIC values (Table S5). We observed an improvement in the goodness of fit between the model considering a temperature dependence on all the reaction steps (All *E_a_*) and when excluding the dependence from the substrate association and product binding steps (No *E_a,on_* and *E_a,bin_* column in Table S5). Additionally, we noticed an improvement of the parameter estimation, as the standard deviations were overall smaller. Thus, considering the improvement in the quality of the fit and on the estimation of the parameters, we continued the process, excluding the temperature dependency of *k_on_* and *k_bin_*. Considering the narrow range of temperatures used for estimating the *E_a_* values, the Arrhenius equation behaves linearly, which could disregard any temperature dependences of *E_a_* [47]. Based on the results obtained, we could claim that, despite the increase in the dissociation rate, increasing the temperature would favor a faster generation of signal. Nevertheless, as can be seen in Figure 5C, at 60 °C, the dissociation rate is comparable to the cleavage rate. Hence, any further increase in the temperature would negatively affect the cleaving capabilities of the DNAzyme, probably caused by approximation to the Tm of the ES complex, which would promote the dissociation of the complex.

Subsequently, to benefit from all the additional information among the temperatures tested, we re-estimated *k_ref_* of the different reaction steps using the Arrhenius equation and the *E_a_* estimated. Additionally, we estimated the relative error of the Substrate_Lg_ and DNAzyme_Lg_ concentration caused by the manual preparation of the reaction samples. As can be seen in Figure 5B, the developed model was able to properly fit the experimental curves obtained at the different temperatures. Then, using bootstrapping, we determined the 95% CI of the *k_ref_*, the *E_a_* parameters and their actual distribution (Table S6); the correlation among the estimates (Figure S6); and the distribution of the errors (Figure S7).

The impact of the temperature on the rate-limiting step was studied by calculating the rate constant of each reaction step for the different temperatures using the Arrhenius equation and the parameters estimated during the bootstrapping process. Then, we performed the sensitivity analysis for the five temperatures included in the model (Figures S8–S12). From the results obtained, we could see that at lower temperatures, the system was more sensitive to changes in the rate constants. Nevertheless, similar to the previous results, *k_off_* was the most robust parameter across the whole temperature and substrate concentration range. Regarding *k_rls_* and *k_bin_*, we noticed that the higher the temperature, the less sensitive the reaction was to these parameters, which could be explained from the DNA hybridization point of view, as the temperature was well above the Tm of the EP complex, promoting, as such, the dehybridization of the strands. Interestingly, regarding *k_on_* and *k_clv_*, we observed that *k_clv_* showed to be the most sensitive parameter for all the temperatures. However, at higher temperatures, we noticed that for lower concentrations of Substrate_Lg_ (100 nmol/L), the reaction was mainly limited by *k_on_*. A possible explanation for these results may be related to the opposite behavior of *k_on_* and *k_clv_* with the temperature. Thus, for low concentrations of substrate from a certain temperature onwards, *k_clv_* becomes larger than *k_on_*, resulting in the latter being able to become the rate-determining rate constant. Additionally, the differences depending on the substrate concentration might be related to the fact that the hybridization step is a first-order reaction, whereas the cleavage step is a zero-order reaction.

### 2.5. Magnesium Dependence

**Figure 6:**
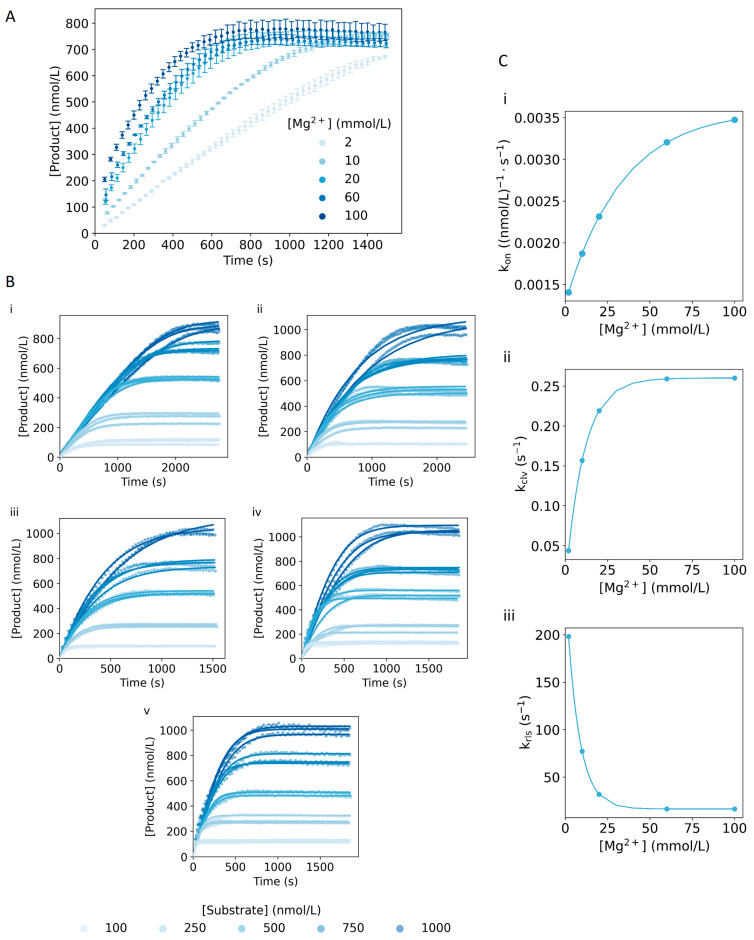
Dependence on the concentration of Mg^2+^. (**A**) Generation of product over time for 10 nmol/L DNAzyme_Lg_ and 750 nmol/L of Substrate_Lg_ for several concentrations of Mg^2+^ (2–100 mmol/L) and at 55 °C. The error bars represent the standard deviation from three independent repetitions. (**B**) Comparison of the product generation in the experimental curves (circles) and the model (solid lines) for different concentrations of magnesium cation: (i) 2 mmol/L, (ii) 10 mmol/L, (iii) 20 mmol/L, (iv) 60 mmol/L and (v) 100 mmol/L. The reaction was measured for 10 nmol/L of DNAzyme_Lg_ and several Substrate_Lg_ concentrations (100–1000 nmol/L) at 55 °C. The parameters estimated can be found in Table S8. (**C**) Scatter plot depicting the relationship between *k_on_* (i), *k_clv_* (ii) and *k_rls_* (iii) with the concentration of Mg^2+^.

The 10–23 DNAzyme was selected in the presence of Mg^2+^, and, as such, its activity depends on the concentration of this cation [8]. In order to study the Mg^2+^ dependence of the rate constants, we monitored the DNAzyme reaction at additional concentrations of Mg^2+^ (0, 2, 10, 60 and 100 mmol/L). An absence of Mg^2+^ resulted in no product being generated, while for the other concentrations, we observed an overall increase in the velocity of the reaction when increasing the concentration of Mg^2+^ (Figure 6A). Additionally, Mg^2+^ being a positive ion, it stabilizes the interaction between two DNA strands [48], influencing also the rate of association and dissociation of the DNAzyme with the substrate and the product. Such relationships were implemented in the model using an exponential function (Equations (9) and (10)), which was selected taking into account the following: (1) an initial optimization we performed by estimating the rate constants for each Mg^2+^ concentration; (2) the fact that we could still observe if the dependence was in the linear range and adjust it accordingly; and (3) the limited number of binding spots on the DNAzyme sequence for the Mg^2+^ to interact with, thus reaching a saturation level.

From the initial model optimization, we could observe a very small dependence of *k_off_* and *k_bin_* on the concentration of Mg^2+^. With the aim of simplifying the model, we removed the dependency on these parameters and optimized the model again, leading to an improvement of the goodness-of-fit parameters and the estimated values, as can be seen in Table S7. Intermediate steps were also evaluated (no dependency on *k_off_* or *k_bin_*, not shown), which resulted in a decrease in the quality of the fit. Therefore, we decided to continue the fitting, excluding the Mg^2+^ dependence on *k_off_* and *k_bin_*. Next, following the methodology detailed in the corresponding section of Materials and Methods and Equations (9) and (10), the 95% CI of the parameters for the exponential functions (Table S8), the correlation among the parameters (Figure S13) and the distribution of errors on the DNAzyme_Lg_ and Substrate_Lg_ concentration were obtained (Figure S14). As shown in Figure 6B, the implemented Mg^2+^ dependence on the reaction steps yielded a model able to rigorously fit the kinetic curves obtained at different cation concentrations. For a deeper analysis of the dependence under study, we calculated and plotted the rate constants against the concentration of cofactor. As can be observed in Figure 6C, *k_rls_* reached a saturation at much lower concentrations of Mg^2+^ than *k_on_* and *k_clv_*, which were still in the more linear part. Focusing on *k_rls_* and taking into account the difference between the concentrations measured in the lowest range, we could not discern between a gradual change or a switch between the two extreme values. Nevertheless, from the point of view of implementing this reaction in, for example, a biosensor, such low concentrations of Mg^2+^ would not be contemplated, as they result in low *k_on_* and *k_clv_* values, decreasing the overall rate of the reaction and, as such, negatively affecting the performance of the biosensor.

To further study the effect of the cofactor on the reaction, we simulated the reaction output after applying a change of up to 60% in each rate constant. An important aspect to consider when interpreting the results of the sensitivity analysis is the assumption that the different reaction steps behave independently. Thus, as can be observed in Figures S15–S19, *k_rls_* was a parameter with high sensitivity across the concentration range studied, especially at low concentrations of Mg^2+^. A possible explanation for these results could be the fact that if the release of the products is slower, the DNAzyme is not able to start a new reaction cycle, affecting in this manner the overall reaction velocity. Moreover, we could observe that for larger ratios between the DNAzyme and the substrate, and when increasing the concentrations of Mg^2+^, inhibition due to rehybridization of the products could affect the reaction. Taking all the observations into account, we could infer that in Mg^2+^ rich environments, the reaction could be limited by interactions between the DNA strands rather than the cleavage step.

### 2.6. Model Implementation for DNAzyme with Shorter Substrate-Binding Arm Sequences

**Figure 7:**
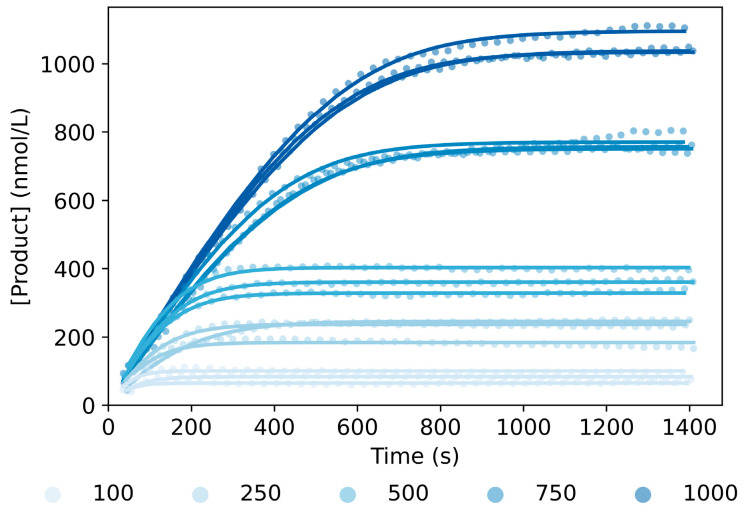
Product generation measured experimentally (symbols) and fitted curves (solid lines) for several concentrations of Substrate_Sh_ (100–1000 nmol/L), 10 nmol/L of DNAzyme_Sh_ and 20 mmol/L of Mg^2+^ at 23 °C. For each substrate concentration, there were three independent repetitions. All experimental curves were fitted simultaneously, using the No Ligation model described in Figure S2B. The parameters estimated can be found in Table S9.

As mentioned earlier, one of the important application fields of DNAzymes is the development of biosensors, which are often desired to perform at room temperature. To study the kinetic behavior of the 10–23 DNAzyme in these conditions, we firstly shortened the sequence of the DNAzyme and the substrate-binding arms by removing four nucleotides on each side (DNAzyme_Sh_ and Substrate_Sh_ in Table S1 in Supplementary Information), following the work by Ven et al. [12]. Then, the product generation was monitored for several Substrate_Sh_ concentrations (100–1000 nmol/L) at 25 °C, in the presence of 20 mmol/L of Mg^2+^. As can be seen by comparing Figure 2 and Figure 7, differences between the two working temperatures become more pronounced for higher substrate concentrations (i.e., 750 and 1000 nmol/L), with the product generation of the shorter DNAzyme at 25 °C being overall faster.

To further characterize the kinetics, the rate constants for the DNAzyme_Sh_ at room temperature were estimated employing the No Ligation model previously selected and using the values from Table S3 (No Ligation column) to start the optimization. As can be seen in Figure 7 (solid lines), the model was able to accurately fit the experimental data obtained for DNAzyme_Sh_, which demonstrates the generic aspect of the model. Based on the estimated rate constants for both DNAzymes (Tables S4 and S9 for the long and short sequences, respectively), we observed that *k_on_* and *k_clv_* were in the same order of magnitude for both DNAzymes. However, for *k_rls_*, we obtained a difference of one order of magnitude between the two sequences, being larger for DNAzyme_Sh_, which indicates that the product sequences are released faster. The results are in agreement with the opposing effect of lowering the temperature and shortening the substrate-binding arms on the stability of a double strand. Thus, the obtained results indicate that shortening the sequence has a greater influence on the rate constant. Moreover, similar to before, the correlation among the rate constants (Figure S20) and the distribution of the errors for the DNAzyme and substrate concentrations (Figure S21) were obtained based on one hundred bootstrap data sets.

Furthermore, to obtain information on the rate-limiting steps, we looked at the significance of each reaction step by simulating the reaction course when applying a change of up to 60% in each rate constant. The results obtained showed that *k_clv_* and *k_on_* were the most sensitive rate constants, in accordance with the results from the DNAzyme_Lg_. Comparing the results obtained for the lowest and highest substrate concentration (Figure S22), we observed that for large ratios between the DNAzyme and the substrate, cleavage of the substrate would be the rate-limiting step. Nevertheless, at lower concentrations of substrate, due to the hybridization step being a first-order reaction, *k_on_* and *k_clv_* have a comparable effect on the overall reaction rate. Therefore, based on the results obtained, we could say that similar to the DNAzyme_Lg_, the catalytic activity of the DNAzyme_Sh_ is determined by *k_clv_* and *k_on_*, and depends on the ratio between the substrate and the DNAzyme.

### 2.7. Implementation of the Model for New Sequences

To facilitate the transferability and use of the presented model by other researchers, we provide a few guidelines and aspects to take into account when implementing the model. In order to estimate the kinetic rate constants for new DNAzyme sequences or new experimental conditions, it would be necessary to have experimental data of a minimum of four substrate concentrations distributed between 100 and 1000 nmol/L and at least three repetitions. The more repetitions, the more information for the model to estimate the rate constants. If Monte Carlo simulations are of interest, at least one additional concentration of substrate, not included in the optimization data set, is needed for the validation. A link to download the software and the script files can be found in the Data Availability section.

An additional aspect to take into account is the fact the model has been validated using certain experimental conditions; thus, any new temperature or Mg^2+^ tested must be within the ranges mentioned in the manuscript. Nevertheless, conditions outside the range for optimization can be tested, but it is not guaranteed that the model can accurately explain them.

## 3. Materials and Methods

### 3.1. Reagents

The oligonucleotides used in this work, summarized in Table S1 in SI, were obtained from Integrated DNA Technologies (IDT, Leuven, Belgium) and used without further purification. Potassium chloride (KCl) was obtained from Acros Organics (Geel, Belgium). Magnesium chloride solution (MgCl_2_) was purchased from Fisher Scientific (Leicestershire, England), and trident Tris-HCl (pH 8.8) was obtained from Genetex Inc. (Irvine, CA, USA). Tris-ethylenediaminetetraacetic acid (TE, 100×) was acquired from Sigma-Aldrich (St. Louis, MO, USA). The pH of the buffers was adjusted using hydrochloric acid (HCl) from Sigma-Aldrich (St. Louis, MO, USA). TE was used to prepare the working solutions of all DNA sequences after being diluted 100×. All dilutions were prepared using UltraPure distilled water (DNAse-RNAse free, Invitrogen, Carlsbad, CA, USA), unless stated otherwise. All reaction mixes were prepared in DNA LoBind tubes (Eppendorf, Hamburg, Germany), pre-heated at the working temperature in a thermomixer (Eppendorf, Hamburg, Germany), and the fluorescence signal was measured in 384-well black–clear bottom microplates (Glasatelier Saillart, Meerhout, Belgium) using the SpectraMax ID3 microplate reader (Molecular Devices LLC, San Jose, CA, USA).

Unless stated otherwise, all measurements were performed in 10 mmol/L Tris-HCl containing 50 mmol/L KCl and 20 mmol/L MgCl_2_ (pH 8.3) at 55 °C. Moreover, all the concentrations of different reaction elements refer to the concentration in the final volume, unless stated otherwise.

### 3.2. Activity Measurements

The cleavage activity of the DNAzyme was measured in 25 µL containing 10 nmol/L of the long or short DNAzyme (DNAzyme_Lg_ and DNAzyme_Sh_ in Table S1 in Supplementary Information) and several concentrations (100, 250, 500, 750 and 1000 nmol/L) of long or short substrate (Substrate_Lg_ or Substrate_Sh_ in Table S1 in Supplementary Information), which is dually labeled with F on the 5′ end and Q on the 3′ end. The activity of the DNAzyme was monitored on a microplate reader, measuring the fluorescence signal every 30 s for at least 25 min, using 485 nm and 535 nm as excitation and emission wavelengths, respectively. Before the measurement, all solutions were incubated for 15 min at the working temperature. Each substrate concentration was measured separately, and the delay between the substrate addition and the start of the measurement was taken into account. Additionally, when adding the substrate, 10 s were timed in between each repetition.

A solution containing only the substrate was used as a negative control when building a calibration curve, which was fitted with a linear regression. In this context, the baseline corresponding to only the substrate signal was calculated for each concentration and subtracted from the reaction curves. Then, a calibration curve was made using the fluorescence intensity at the plateau of the reaction curves; the slope of the linear regression was used to normalize the fluorescence intensity.

### 3.3. Derivation of the ODEs

The equations used to fit the kinetic curves were derived based on the reaction mechanism depicted in Figure 1 and by implementing the law of mass action for each reaction element (Equations (1)–(5)). An overview of the units for the different equation parameters can be found in Table S2.

(1) $$\frac{d\left[S\right]}{dt}=-k_{on}\cdot \left[S\right]\cdot \left[E\right]+k_{off}\cdot \left[ES\right]$$

(2) $$\frac{d\left[E\right]}{dt}=-k_{on}\cdot \left[S\right]\cdot \left[E\right]+k_{off}\cdot \left[ES\right]+k_{rls}\cdot \left[EP\right]-k_{bin}\cdot \left[E\right]\cdot \left[P\right]$$

(3) $$\frac{d\left[ES\right]}{dt}=k_{on}\cdot \;\left[S\right]\cdot \left[E\right]-k_{off}\cdot \left[ES\right]-k_{clv}\cdot \left[ES\right]+k_{lig}\cdot \left[EP\right]$$

(4) $$\frac{d\left[EP\right]}{dt}=k_{clv}\cdot \left[ES\right]-k_{lig}\cdot \left[EP\right]-k_{rls}\cdot \left[EP\right]+k_{bin}\cdot \;\left[E\right]\cdot \left[P\right]$$

(5) $$\frac{d\left[P\right]}{dt}=k_{rls}\cdot \left[EP\right]-k_{bin}\cdot \left[E\right]\cdot \left[EP\right]$$

where the association, dissociation, cleavage, ligation, release and binding rate constants are defined by *k_on_*, *k_off_*, *k_clv_*, *k_lig_*, *k_rls_* and *k_bin_*, respectively.

### 3.4. Model Optimization

#### 3.4.1. Model Selection and Optimization

The reaction curves were fitted with Equations (1)–(5) using an in-house developed software, OptiPa v6.2p [49]. The optimization process consisted of several steps. Firstly, an enhanced scatter search (ESS) [50] was performed by scanning two orders of magnitude around the initial values. Secondly, the estimates of the scatter search were used as initial values for an optimization with the lsqnonlin method. For the calculation of the root-mean-square error (RMSE), the sum of the concentration of EP and P was used as the predicted value. Upon selection of the final model structure, the experimental error originating from pipetting variabilities was accounted for in the concentration of substrate and DNAzyme by introducing the terms ${Err}_{E}$ and ${Err}_{S}$, respectively.

(6) $$S\left(0\right)=Err_{S}\;\cdot \;{\bar{S}}_{0}$$

(7) $$E\left(0\right)=Err_{E}\;\cdot {\bar{E}}_{0}$$

Keeping the rate constants at a fixed value, the error terms were estimated for each experimental curve separately and used to initiate the state variables (Equations (6) and (7)), also using the lsqnonlin method. Finally, a last optimization step was performed estimating the kinetic parameters and the error terms together.

#### 3.4.2. CI Determination

The 95% CI of the model parameters were obtained by creating 100 bootstrap data sets based on the original data set, using the error-based re-sampling technique implemented in OptiPa. For each bootstrap data set, the rate constants and the error terms were estimated, using the parameter values estimated for the original data set as initial values. The 95% CI of each parameter was determined by OptiPa, from the distribution of the 100 sets of parameter estimates on the individual bootstrap data sets.

#### 3.4.3. Monte Carlo Simulation of the Model Output

The calculation of the model output based on the variability of the *Err_E_* and *Err_S_* was performed by means of Monte Carlo simulations, using OptiPa. The CIs of ${Err}_{E}$ and ${Err}_{S}$ obtained for each experimental curve in the previous step were combined in a unique set for ${Err}_{E}$ and ${Err}_{S}$. Next, a random set of 1000 combinations of both errors was drawn from their corresponding distributions and used together with the estimated values of the rate constants to simulate the output of the reaction for the specified substrate and DNAzyme concentrations. Then, the probability density functions for the simulated outputs were computed.

### 3.5. Temperature Dependence

The kinetic rate constants were assumed to depend on temperature following the Arrhenius law (Equation (8)).

(8) $$k=k_{ref}\cdot e^{\frac{E_{a}}{R}\;\cdot \;\left(\frac{1}{T_{ref}}-\frac{1}{T}\right)}$$

where *k* is the rate constant at the temperature of interest (*T*) and *k_ref_* the rate constant at the reference temperature (*T_ref_*). *E_a_* refers to the activation energy, and *R* is the universal gas constant. The estimation of the parameters was achieved by performing an initial ESS of two orders of magnitude, followed by a lsqnonlin optimization of *E_a_* while keeping *k_ref_* as constants. After the final selection of the model structure and estimation of the *E_a_* values, the *k_ref_* values were estimated again using the data for all temperatures. Then, the errors on the DNAzyme and substrate concentrations were determined, followed by the calculation of the 95% CI using the bootstrap data sets.

### 3.6. Magnesium Dependence

The kinetic rate constants were assumed to depend on magnesium in an exponential fashion, either decreasingly (Equation (9)) or increasingly (Equation (10)).

(9) $$k=a+b\cdot e^{-c\left[\mathrm{Mg}\right]}$$

(10) $$k=a+b\cdot \left(1-e^{-c\left[\mathrm{Mg}\right]}\right)$$

where *k* is the rate constant at the Mg^2+^ concentration of interest ([Mg]). Equation (9) was applied to the rate constants *k_off_* and *k_rls_*, while Equation (10) was applied to *k_on_*, *k_clv_* and *k_bin_*. In the case of *k_clv_*, taking into account that the DNAzyme is not able to cleave in the absence of Mg^2+^, the y-intercept was forced to zero by implying *a* = 0. The fitting of all the parameters was conducted with an ESS of one order of magnitude, succeeded by a lsqnonlin optimization step. Next, another fitting was performed to estimate the errors on the DNAzyme and substrate.

## 4. Conclusions

In this work, we have developed a mathematical model capable of describing and predicting the cleavage reaction catalyzed by the 10–23 DNAzyme under several reaction conditions. To achieve this, we have established a reaction mechanism based on literature information and used the law of mass action to derive the kinetic equations. Starting from the full equilibrium on all the reaction steps, we determined that the ligation could be excluded from the model, resulting in a better fit of the experimental data. Next, we tested the feasibility of the model to predict the outcome of the reaction, based on the variability of the Substrate_Lg_ and DNAzyme_Lg_ concentrations using Monte Carlo simulations, which resulted in an outstanding agreement between the experimental and simulated curves for a concentration of Substrate_Lg_ up to 1000 nmol/L. Furthermore, we demonstrated the usefulness of the model to analyze how the experimental conditions affect each individual reaction step. In this context, we studied the dependence of the rate constants on the temperature using the Arrhenius equation, observing that *k_off_*, *k_clv_* and *k_rls_* are significantly more sensitive to temperature changes. The relation obtained was included in the model, thereby becoming temperature independent. Similarly, we implemented an exponential dependence of the kinetic rate constants on Mg^2+^ as the reaction cofactor, which showed saturation at low Mg^2+^ concentrations for *k_rls_*, but a fairly linear relation for *k_on_* and *k_clv_* within the range studied. We also investigated the impact of lowering the working temperature and shortening the substrate-binding arms. Additionally, we used the model to evaluate the sensitivity of the reaction rates and identify the rate-limiting step. Although there is not a unique answer, the results obtained indicated the cleavage and association steps as main determinants of the overall reaction speed. Moreover, when changing the experimental conditions, additional rate constants, such as *k_rls_*, became more relevant. Therefore, under multiple turnover conditions, the cleavage activity of the DNAzyme is not only determined by the catalytic core, but the association/dissociation kinetics of the substrate and product sequences are also important factors. Accordingly, differences in cleavage activity among DNAzymes with different compositions of the substrate-binding arms could be mostly explained by the rate constants referring to the hybridization kinetics between the substrate and the DNAzyme.

The results shown in this work reveal the potential of the mathematical model to understand and characterize an enzymatic reaction. In the case of the 10–23 RNA-cleaving DNAzymes, this tool is extremely interesting for both diagnostic and therapeutic applications, as it would allow for predicting whether specific experimental conditions are viable without having to perform an extensive set of experiments. Consequently, the process would benefit from a reduction in the time and cost of optimization. Additionally, this work depicts the premises of the numerous possibilities that could be exploited with mathematical models. By providing guidelines to implement the presented model, we aim to facilitate its use for researchers and its further development. Further research could elaborate on the experimental conditions included in the model, such as pH, different metal cations or the composition of the substrate-binding arms. In addition, the combined effect of different reaction conditions, such as temperature and Mg^2+^, would also be an interesting subject to study, as it would widen the applicability of the model.

## Figures and Tables

**Table 1:** Overview of the rate constants for the DNAzyme_Lg_ catalyzed reaction and the 95% confidence interval (CI) determined from 100 randomly generated bootstrap data sets.

Parameter	95% CI
*k_on_* ((nmol/L)^−1^·s^−1^)	4.32 × 10^−3^–4.73 × 10^−3^
*k_off_* (s^−1^)	1.46 × 10^−1^–1.65 × 10^−1^
*k_clv_* (s^−1^)	2.10 × 10^−1^–2.34 × 10^−1^
*k_rls_* (s^−1^)	1.91 × 10^1^–2.22 × 10^1^
*k_bin_* ((nmol/L)^−1^·s^−1^)	1.07 × 10^−1^–1.19 × 10^−1^

## Data Availability

OptiPa is a free tool and can be downloaded from www.optipa.be (accessed on 30 August 2023). The script files used in OptiPa can be downloaded from https://github.com/AiidaMP/DNAzyme-kinetics-model (accessed on 30 August 2023). The experimental data are available on request.

## Supplementary Materials

The following are available online at https://www.mdpi.com/article/10.3390/ijms241813686/s1, Supporting information contains the DNA sequences, the overview of parameter estimates and correlation matrixes for the different models, together with the distribution of the error values. Moreover, it includes the Monte Carlo simulations for substrate concentration outside the training range, and the plots for all the conditions evaluated during the sensitivity analysis. Finally, the minimal set of experiments necessary to estimate the rate constants for new sequences is indicated.
