# Supplementary material for: Unraveling the Kinetics of the 10–23 RNA-Cleaving DNAzyme
Source: Int J Mol Sci. 2023 Sep 5;24(18):13686. doi: 10.3390/ijms241813686 (PMC10531344; doi:10.3390/ijms241813686)
Supplement: Supplementary file 1 [file ijms-24-13686-s001.zip › ijms-2474500-supplementary.pdf]

ELECTRONIC SUPLEMENTARY INFORMATION

Unraveling the Kinetics of the 10–23 RNA-Cleaving DNAzyme

Aida Montserrat Pagès[1], Maarten Hertog[2], Bart Nicolaï[2], Dragana Spasic[1] and Jeroen Lammertyn[1*]

[1]*Department of Biosystems, Biosensors Group, KU Leuven, 3001, Leuven, Belgium*

[2]*Department of Biosystems, Postharvest Group, KU Leuven, 3001, Leuven, Belgium*

*corresponding author: jeroen.lammertyn@kuleuven.be

Table S1 Summary of the nucleic acid (NA) sequences used in this study written in 5' – 3' direction. The different parts of DNAzyme are depicted as follows: the catalytic core with the underlined bases and the *substrate-binding arms* with the bases in italics DNA bases are represented in upper case, and RNA bases are represented in lower case. The substrate sequence was modified with a fluorescence reporter (FAM) and a quencher (Iowa Black® FQ quencher, IBFQ).

DNAzyme			
$DNAzyme_{Lg}$	*CGGTTGGTGA*	GGCTAGCTACAACGA	*GGTTGTGCTG*
$DNAzyme_{Sh}$	*TGGTGA*	GGCTAGCTACAACGA	*GGTTGT*
**Substrate**			
$Substrate_{Lg}$	FAM	CAGCACAACCrGrUCACCAACCG	IBFQ
$Substrate_{Sh}$	FAM	ACAACCrGrUCACCA	IBFQ

Table S2 Overview of the parameters of the equations used to describe the reaction and the dependence on the temperature and concentration of Mg^{2+}

Parameter	Units	Parameter	Units
[S]	nmol/L	$E_{a,bin}$	J/mol
[E]	nmol/L	a_{on}	$(nmol/L)^{-1} \cdot s^{-1}$
[ES]	nmol/L	b_{on}	$(nmol/L)^{-1} \cdot s^{-1}$
[EP]	nmol/L	c_{on}	$(nmol/L)^{-1}$
[P]	nmol/L	a_{off}	s^{-1}
k_{on}, $k_{on\text{-}ref}$	$(nmol/L)^{-1} \cdot s^{-1}$	b_{off}	s^{-1}
k_{off}, $k_{off\text{ -}ref}$	s^{-1}	c_{off}	$(nmol/L)^{-1}$
k_{clv}, $k_{clv\text{ -}ref}$	s^{-1}	a_{clv}	s^{-1}
k_{lig}, $k_{lig\text{ -}ref}$	s^{-1}	c_{clv}	$(nmol/L)^{-1}$
k_{rls}, $k_{rls\text{ -}ref}$	s^{-1}	a_{rls}	s^{-1}
k_{bin}, $k_{bin\text{-}ref}$	$(nmol/L)^{-1} \cdot s^{-1}$	b_{rls}	s^{-1}
$E_{a,on}$	J/mol	c_{rls}	$(nmol/L)^{-1}$
$E_{a,off}$	J/mol	a_{bin}	$(nmol/L)^{-1} \cdot s^{-1}$
$E_{a,clv}$	J/mol	b_{bin}	$(nmol/L)^{-1} \cdot s^{-1}$
$E_{a,rls}$	J/mol	c_{bin}	$(nmol/L)^{-1}$

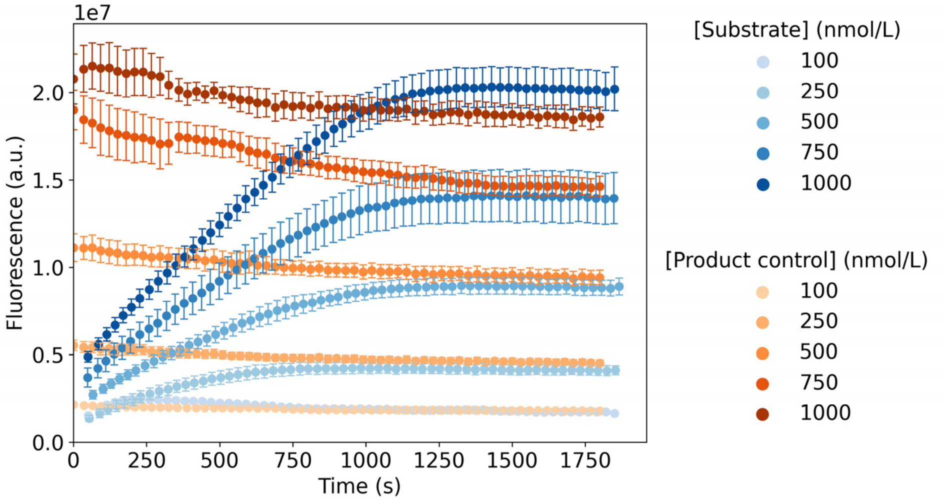

Figure S1 Assessment of full $\text{Substrate}_{\text{Lg}}$ conversion into product. The fluorescence intensity of the cleaved substrate (blue) was compared with the intensity of a fluorescently labelled sequence representing half of the original substrate (orange). The error bars represent the standard deviation of three repetition.

A

$$E + S \underset{k_{off}}{\overset{k_{on}}{\rightleftarrows}} ES \xrightarrow{k_{clv}} EP \underset{k_{bin}}{\overset{k_{rls}}{\rightleftarrows}} E + P$$

B

$$E + S \underset{k_{off}}{\overset{k_{on}}{\rightleftarrows}} ES \xrightarrow{k_{clv}} EP \xrightarrow{k_{rls}} E + P$$

Figure S2 Minimal kinetic scheme of the reaction mechanism variations: **A**) removing the ligation of the product and **B**) excluding both the ligation and the binding of the product sequences.

Table S3 Overview of the rate constant estimates after the optimization of the model considering the sum of the DNAzyme-product (EP) and the product (P) sequences as the output of the model. The model was optimized using the least square non-linear method and different versions were tested, differing in the number of parameters describing the reaction. Additionally, the last two rows depict two statistics to evaluate the goodness of fit, the Akaike's information criterion (AIC) and the root mean square error (RMSE).

	Full equilibrium	No Ligation	No binding
**k_{on} ((nmol/L)$^{-1}$ · s^{-1})**	$1.91 \times 10^{-3} \pm 1.63 \times 10^{-4}$	$4.63 \times 10^{-3} \pm 2.31 \times 10^{-4}$	$8.52 \times 10^{-4} \pm 7.18 \times 10^{-4}$
**k_{off} (s^{-1})**	$2.61 \times 10^{-2} \pm 1.23 \times 10^{-2}$	$1.32 \times 10^{-1} \pm 1.52 \times 10^{-2}$	$8.75 \times 10^{-2} \pm 3.89 \times 10^{0}$
**k_{clv} (s^{-1})**	$2.23 \times 10^{-1} \pm 4.91 \times 10^{-3}$	$2.08 \times 10^{-1} \pm 2.49 \times 10^{-3}$	$4.63 \times 10^{0} \pm 4.99 \times 10^{0}$
**k_{lig} (s^{-1})**	$1.24 \times 10^{-3} \pm 3.97 \times 10^{-3}$		
**k_{rls} (s^{-1})**	$5.96 \times 10^{0} \pm 1.19 \times 10^{0}$	$2.17 \times 10^{1} \pm 1.32 \times 10^{0}$	$2.29 \times 10^{-1} \pm 1.44 \times 10^{-2}$
**k_{bin} ((nmol/L)$^{-1}$ · s^{-1})**	$1.44 \times 10^{-2} \pm 3.23 \times 10^{-3}$	$1.09 \times 10^{-1} \pm 6.94 \times 10^{-3}$	
**AIC**	13272	13307	13626
**RMSE**	25.834	26.062	28.19

Table S4 Summary of the parameters describing the distribution of the rate constants obtained from 100 randomly generated bootstrap data sets. For each experimental curve in the data set, 100 simulated samples were obtained, and the rate constants were estimated for each of them. (CI: Confidence interval)

	Mean	Standard deviation	Lower bound (95 % CI)	Upper bound (95 % CI)	Skewness	Kurtosis
**k_{on} ((nmol/L)$^{-1}$ · s^{-1})**	4.51×10^{-3}	1.18×10^{-4}	4.32×10^{-3}	4.73×10^{-3}	5.59×10^{-1}	5.92×10^{-1}
**k_{off} (s^{-1})**	1.56×10^{-1}	5.54×10^{-3}	1.46×10^{-1}	1.65×10^{-1}	3.71×10^{-1}	7.39×10^{-1}
**k_{clv} (s^{-1})**	2.21×10^{-1}	6.02×10^{-3}	2.10×10^{-1}	2.34×10^{-1}	3.37×10^{-1}	9.81×10^{-1}
**krls (s^{-1})**	2.10×10^{1}	6.87×10^{-1}	1.95×10^{1}	2.22×10^{1}	-1.08×10^{-1}	1.89×10^{-1}
**k_{bin} ((nmol/L)$^{-1}$ · s^{-1})**	1.13×10^{-1}	3.35×10^{-3}	1.07×10^{-1}	1.19×10^{-1}	1.53×10^{-1}	-3.19×10^{-1}

	k_{on}	k_{off}	k_{clv}	k_{rls}	k_{bin}
k_{on}	**1**	-0.21	-0.05	-0.82	0.35
k_{off}	-0.21	**1**	-0.24	0.06	-0.72
k_{clv}	-0.05	-0.24	**1**	-0.08	-0.02
k_{rls}	-0.82	0.06	-0.08	**1**	-0.25
k_{bin}	0.35	-0.72	-0.02	-0.25	**1**

Figure S3 Correlation matrix of the kinetic parameters for the full equilibrium – no ligation model.

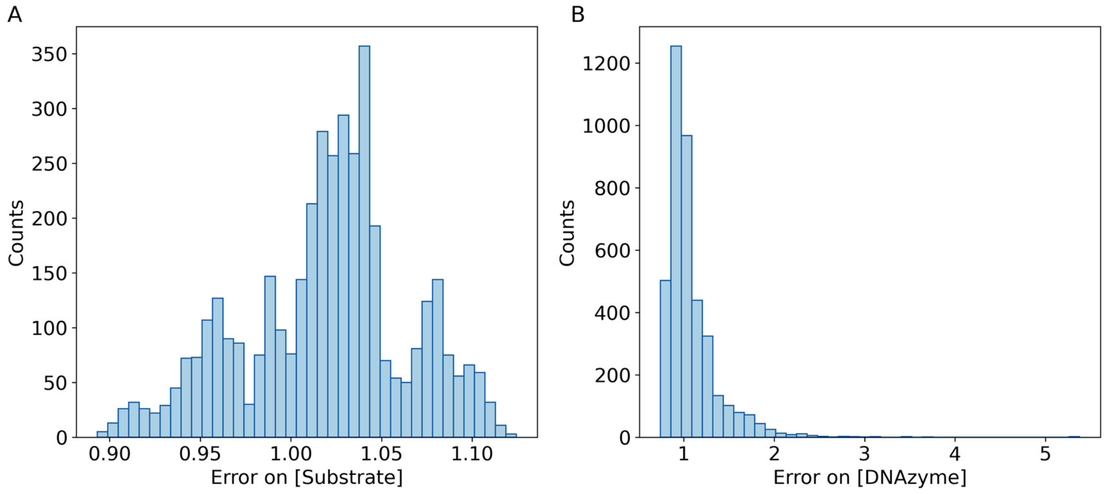

Figure S4 Histogram depicting the distribution of the relative error of the concentration of Substrate$_{Lg}$ **A**) and DNAzyme$_{Lg}$ **B**) obtained during the bootstrapping procedure. For each experimental curve in the data set, 100 simulated samples were obtained, and the errors were estimated for each of them. The histograms have been drawn based on 4000 examples.

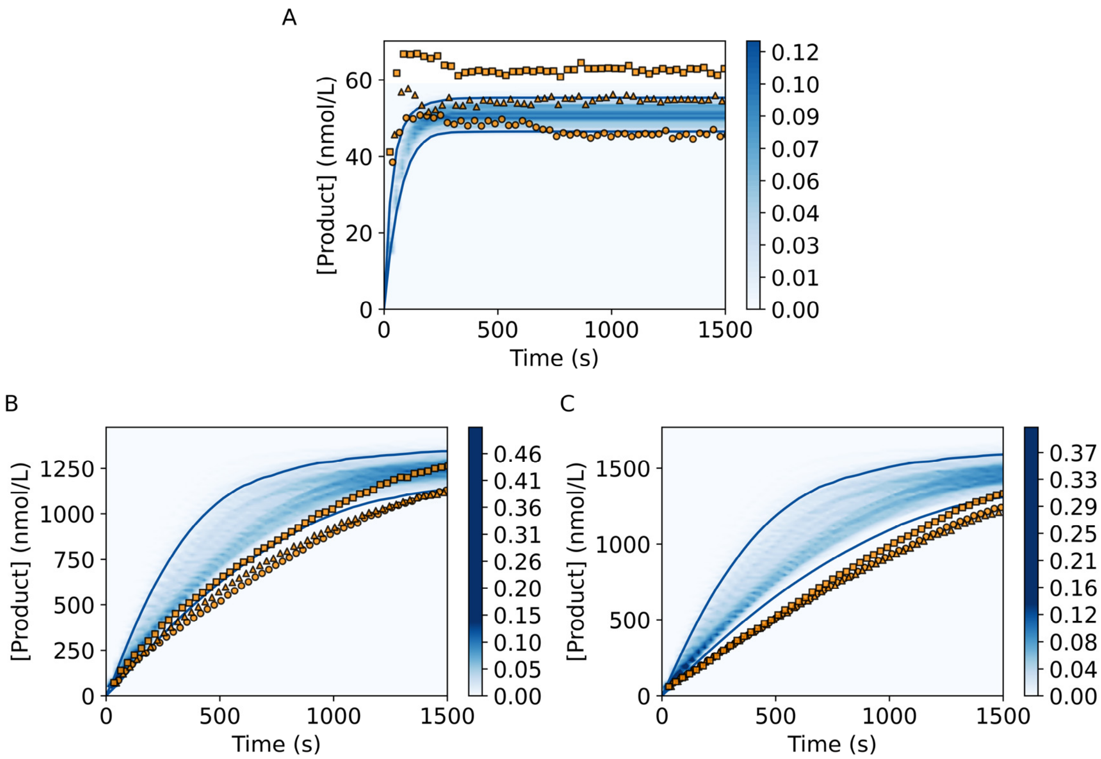

Figure S5 Prediction of the time course of the DNAzyme reaction for 10 nmol/L of DNAzyme and several substrate concentrations: **A**) 50, **B**) 1250 and **C**) 1500 nmol/L. The colormap represents the frequency of the distributions of the modelled product as a function of time, for each time point the sum of all the frequencies equals to 1. The three orange symbols indicate the three validation experiments and the blue solid lines depict the 95 % CI. The reaction has been performed at 55 °C and in the presence of 20 mmol/L of Mg^{2+}.

Table S5 Overview of the estimates and statistics to evaluate the goodness of fit, the Akaike's information criterion (AIC) and the root mean square error (RMSE). The model was optimized using the least square non-linear method and different versions were tested, differing in the number of parameters describing the reaction.

	All Ea	No Ea_{on}	No Ea_{bin}	No Ea_{on} and Ea_{bin}
**Ea_{on} (J/mol)**	$9.97 \times 10^0 \pm 5.36 \times 10^{-1}$		$6.22 \times 10^0 \pm 2.02 \times 10^{-1}$	
**Ea_{off} (J/mol)**	$3.08 \times 10^5 \pm 9.77 \times 10^3$	$3.21 \times 10^5 \pm 5.82 \times 10^3$	$3.58 \times 10^5 \pm 6.95 \times 10^3$	$3.08 \times 10^5 \pm 5.31 \times 10^3$
**Ea_{clv} (J/mol)**	$1.03 \times 10^5 \pm 3.18 \times 10^3$	$1.16 \times 10^5 \pm 2.27 \times 10^3$	$1.19 \times 10^5 \pm 2.78 \times 10^3$	$1.03 \times 10^5 \pm 2.28 \times 10^3$
**Ea_{rls} (J/mol)**	$2.74 \times 10^5 \pm 5.19 \times 10^3$	$2.56 \times 10^5 \pm 4.56 \times 10^3$	$2.57 \times 10^5 \pm 5.63 \times 10^3$	$2.74 \times 10^5 \pm 4.80 \times 10^3$
**Ea_{bin} (J/mol)**	$9.97 \times 10^0 \pm 2.89 \times 10^{-1}$	$1.35 \times 10^1 \pm 2.18 \times 10^{-1}$		
**RMSE**	44.210	44.145	43.996	44.201
**AIC**	35400	35386	35354	35396

Table S6 Summary of the parameters describing the distribution of the rate constants obtained during the bootstrapping procedure. For each experimental curve in the data set, 100 randomly generated bootstrap data sets were obtained, and the rate constants were estimated for each of them. The reference temperature is 55 °C.

	Mean	Standard deviation	Lower bound (95 % CI)	Upper bound (95 % CI)	Skewness	Kurtosis
**$k_{on\text{-}ref}$ ((nmol/L)$^{-1}$ · s^{-1})**	5.84×10^{-3}	1.71×10^{-4}	5.49×10^{-3}	6.15×10^{-3}	7.22×10^{-2}	-3.38×10^{-1}
**$k_{off\text{-}ref}$ (s^{-1})**	1.48×10^{-1}	7.46×10^{-3}	1.31×10^{-1}	1.62×10^{-1}	7.61×10^{-2}	3.16×10^{-2}
**Ea_{off} (J/mol)**	2.29×10^{5}	8.68×10^{3}	2.11×10^{5}	2.46×10^{5}	1.84×10^{-1}	6.63×10^{-1}
**$k_{clv\text{-}ref}$ (s^{-1})**	2.50×10^{-1}	7.94×10^{-3}	2.33×10^{-1}	2.65×10^{-1}	-2.60×10^{-2}	-2.89×10^{-2}
**Ea_{clv} (J/mol)**	1.39×10^{5}	8.13×10^{3}	1.23×10^{5}	1.56×10^{5}	-1.07×10^{-1}	8.57×10^{-1}
**$k_{rls\text{-}ref}$ (s^{-1})**	1.68×10^{1}	5.91×10^{-1}	1.55×10^{1}	1.79×10^{1}	5.59×10^{-2}	1.84×10^{-1}
**Ea_{rls} (J/mol)**	3.37×10^{5}	6.44×10^{3}	3.24×10^{5}	3.47×10^{5}	8.76×10^{-2}	1.74×10^{0}
**$k_{bin\text{-}ref}$ (s^{-1})**	8.61×10^{-2}	2.51×10^{-3}	8.07×10^{-2}	9.21×10^{-2}	1.01×10^{-1}	8.74×10^{-1}

	$k_{on\text{-}ref}$	$k_{off\text{-}ref}$	Ea_{off}	$k_{clv\text{-}ref}$	Ea_{clv}	$k_{rls\text{-}ref}$	Ea_{rls}	$k_{bin\text{-}ref}$
**$k_{on\text{-}ref}$**	**1**	-0.05	0.1	0.1	0.13	-0.55	0.05	0.39
**$k_{off\text{-}ref}$**	-0.05	**1**	-0.42	-0.01	-0.25	0.41	-0.08	-0.3
**Ea_{off}**	0.1	-0.42	**1**	0.09	-0.13	-0.41	0.22	-0.24
**$k_{clv\text{-}ref}$**	0.1	-0.01	0.09	**1**	-0.36	0.38	-0.2	-0.35
**Ea_{clv}**	0.13	-0.25	-0.13	-0.36	**1**	-0.45	0.59	0.19
**$k_{rls\text{-}ref}$**	-0.55	0.41	-0.41	0.38	-0.45	**1**	-0.29	-0.17
**Ea_{rls}**	0.05	-0.08	0.22	-0.2	0.59	-0.29	**1**	-0.32
**$k_{bin\text{-}ref}$**	0.39	-0.3	-0.24	-0.35	0.19	-0.17	-0.32	**1**

Figure S6 Correlation matrix of the kinetic parameters (k) and the activation energies (Ea) for the temperature model.

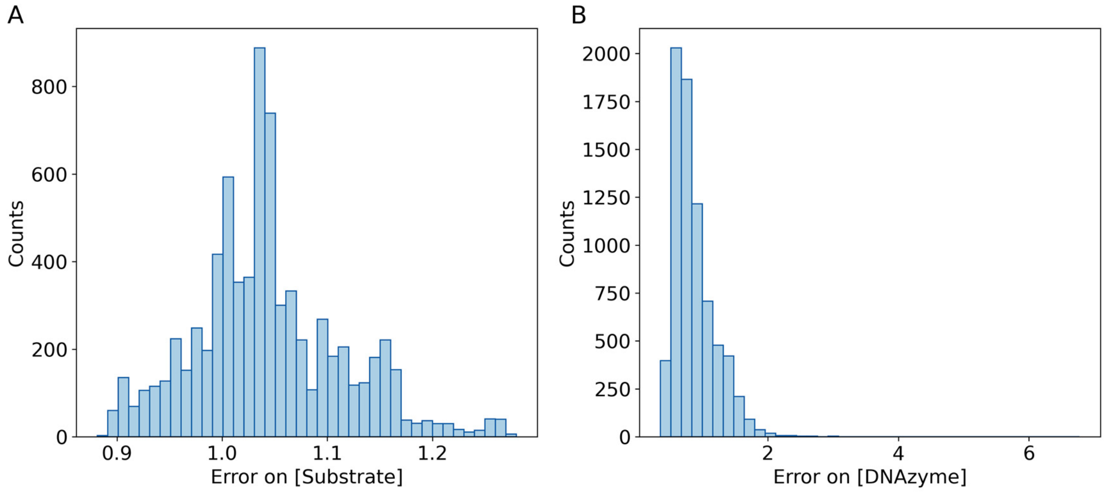

Figure S7 Histogram depicting the distribution of the relative error of the concentration of $\text{Substrate}_{\text{Lg}}$ **A**) and $\text{DNAzyme}_{\text{Lg}}$ **B**) obtained during the bootstrapping procedure. For each experimental curve in the data set, 100 simulated samples were obtained, and the errors were estimated for each of them. The histograms have been drawn based on 7500 examples.

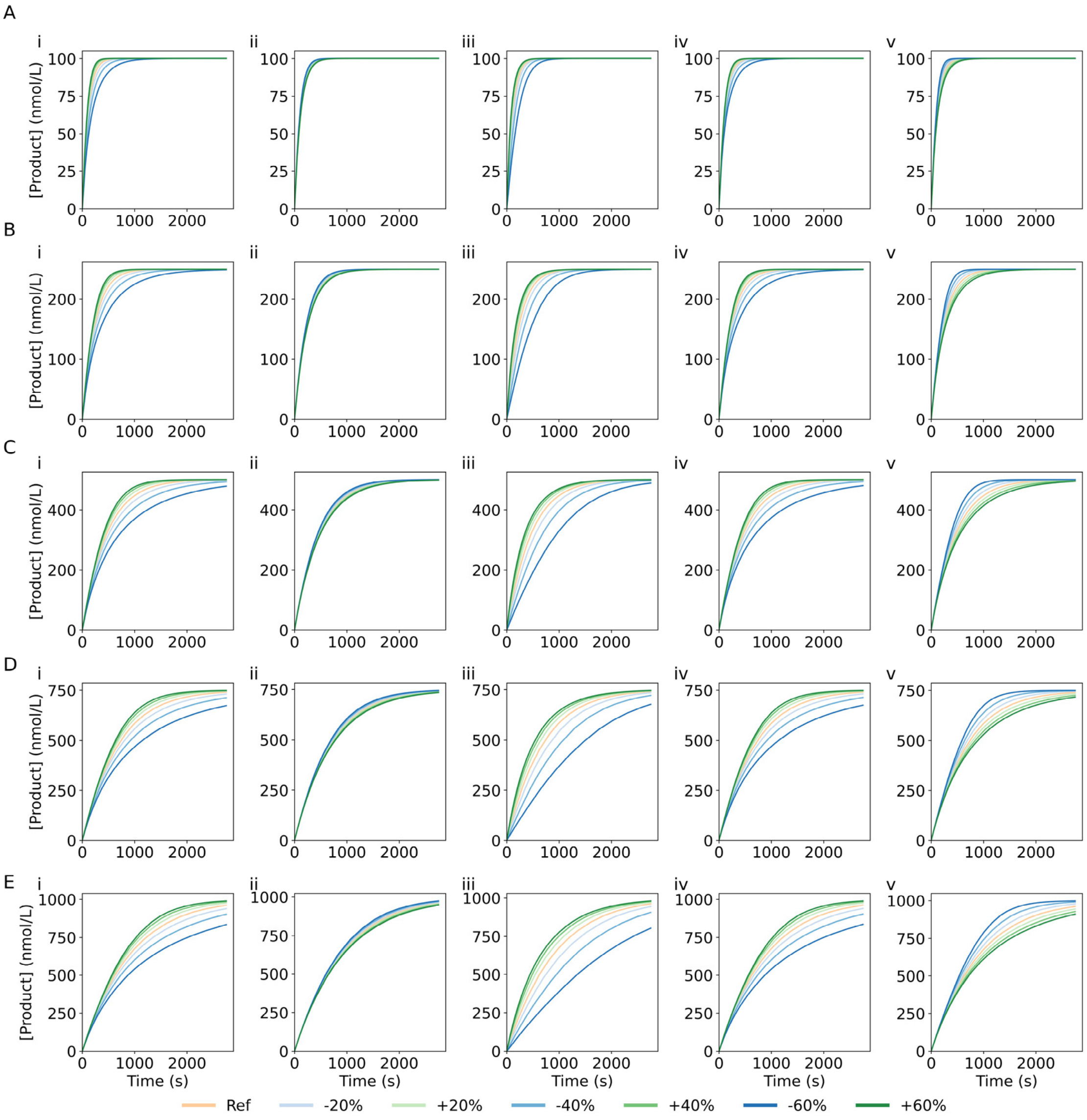

Figure S8 Simulation of the model output at 50 °C when altering the kinetic rate constants for the different Substrate$_{Lg}$ concentrations: **A**) 100, **B**) 250, **C**) 500, **D**) 750 and **E**) 1000 nmol/L. To perform this analysis, the rate constants were systematically evaluated in a range of ± 60 % calculated from the estimate values shown in Table S6. The process was performed for each of the rate constant independently: (i) k_{on}, (ii) k_{off}, (iii) k_{clv}, (iv) k_{rls} and (v) k_{bin}.

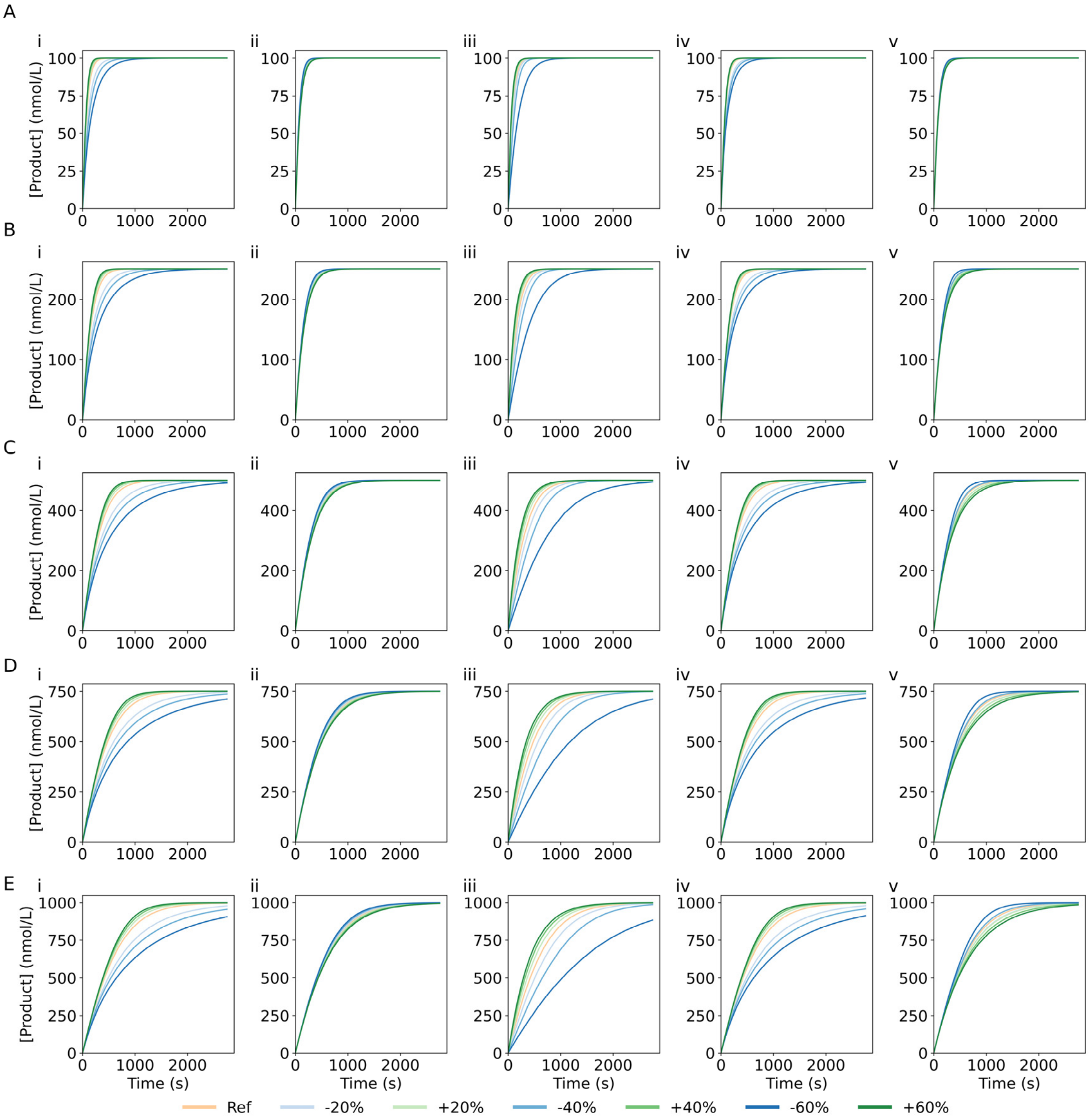

Figure S9 Simulation of the model output at 52 °C when altering the kinetic rate constants for the different $\text{Substrate}_{\text{Lg}}$ concentrations: **A**) 100, **B**) 250, **C**) 500, **D**) 750 and **E**) 1000 nmol/L. To perform this analysis, the rate constants were systematically evaluated in a range of ± 60 % calculated from the estimate values shown in Table S6. The process was performed for each of the rate constant independently: (i) k_{on}, (ii) k_{off}, (iii) k_{clv}, (iv) k_{rls} and (v) k_{bin}.

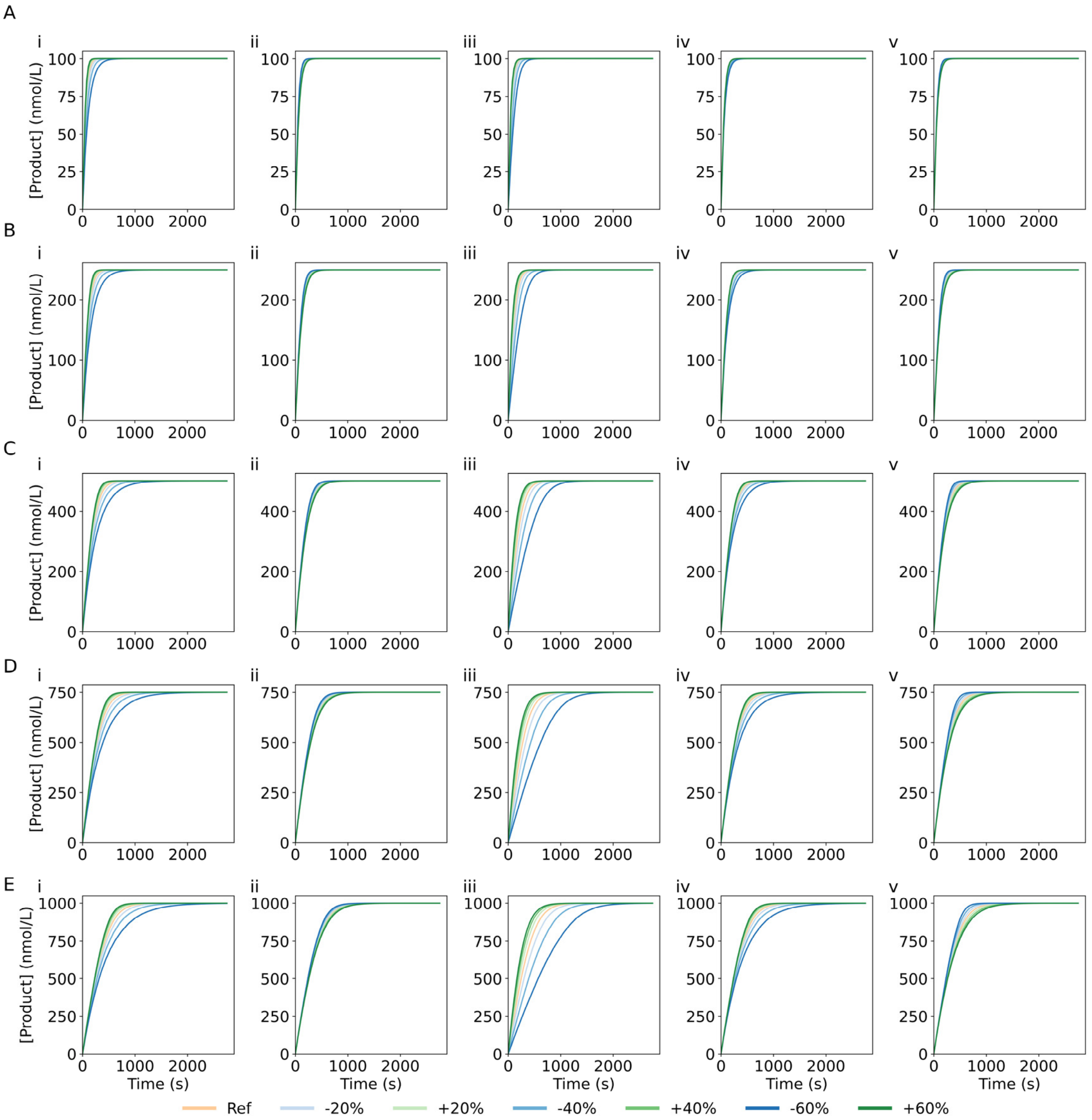

Figure S10 Simulation of the model output at 55 °C when altering the kinetic rate constants for the different Substrate$_{Lg}$ concentrations: **A**) 100, **B**) 250, **C**) 500, **D**) 750 and **E**) 1000 nmol/L. To perform this analysis, the rate constants were systematically evaluated in a range of ± 60 % calculated from the estimate values shown in Table S6. The process was performed for each of the rate constant independently: (i) k_{on}, (ii) k_{off}, (iii) k_{clv}, (iv) k_{rls} and (v) k_{bin}.

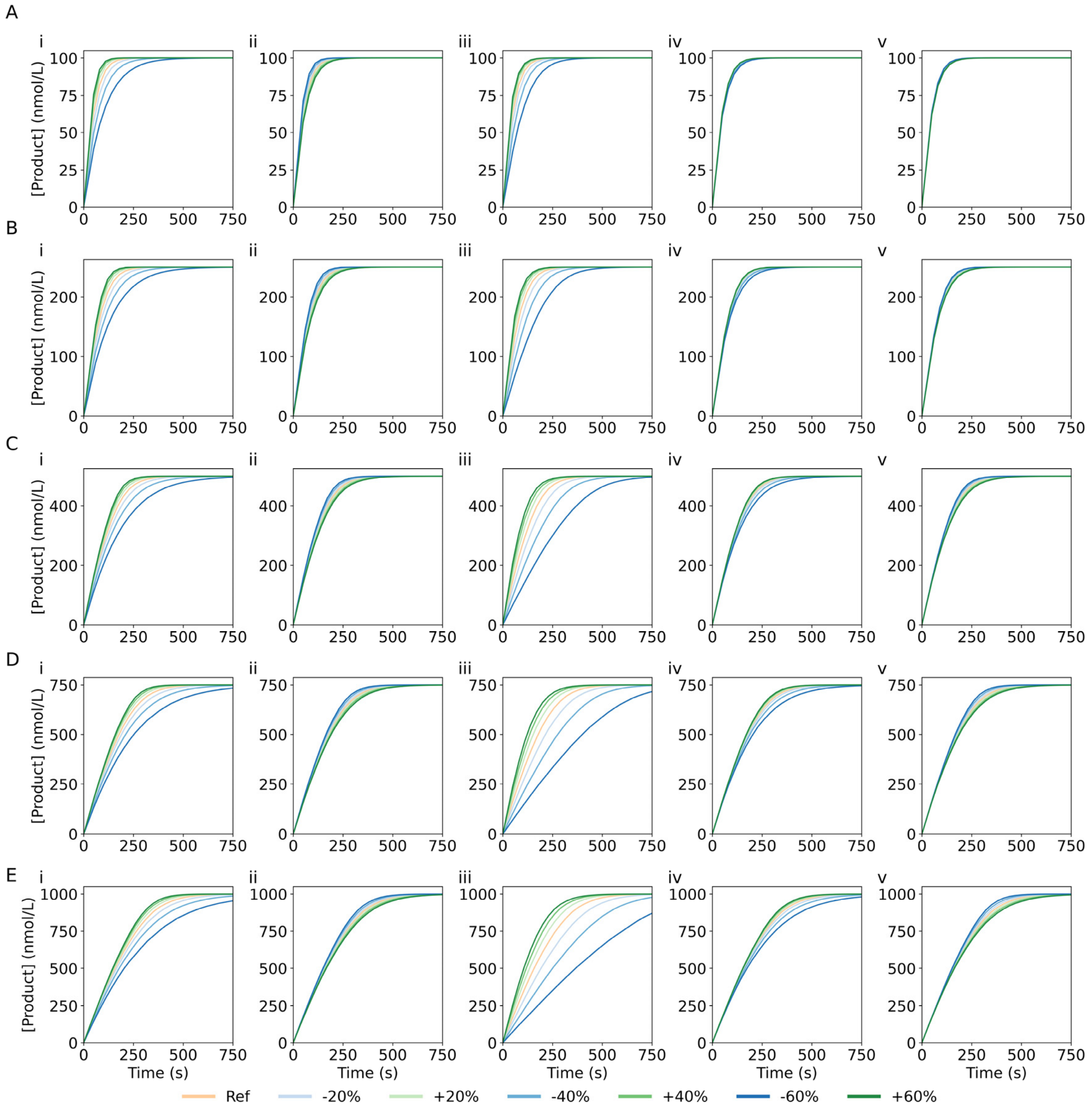

Figure S11 Simulation of the model output at 58 °C when altering the kinetic rate constants for the different Substrate_{Lg} concentrations: **A**) 100, **B**) 250, **C**) 500, **D**) 750 and **E**) 1000 nmol/L. To perform this analysis, the rate constants were systematically evaluated in a range of ± 60 % calculated from the estimate values shown in Table S6. The process was performed for each of the rate constant independently: (i) k_{on}, (ii) k_{off}, (iii) k_{clv}, (iv) k_{rls} and (v) k_{bin}. For visualization purposes the x-axis shows until 750 s.

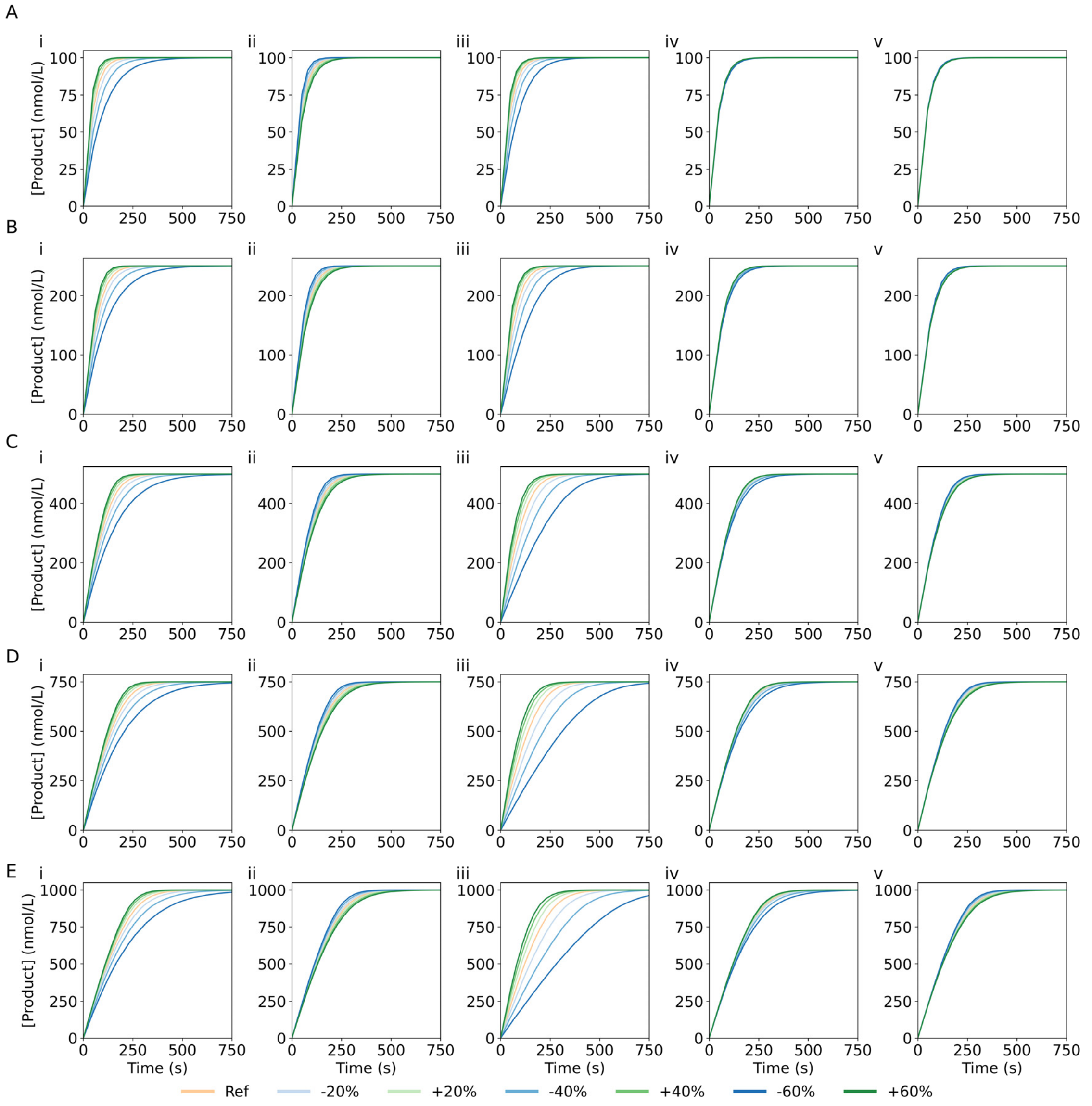

Figure S12 Simulation of the model output at 60 °C when altering the kinetic rate constants for the different $Substrate_{Lg}$ concentrations: **A**) 100, **B**) 250, **C**) 500, **D**) 750 and **E**) 1000 nmol/L. To perform this analysis, the rate constants were systematically evaluated in a range of ± 60 % calculated from the estimate values shown in Table S6. The process was performed for each of the rate constant independently: (i) k_{on}, (ii) k_{off}, (iii) k_{clv}, (iv) k_{rls} and (v) k_{bin}. For visualization purposes the x-axis shows until 750 s.

Table S7 Overview of the exponential function estimates and the statistics to evaluate the goodness of fit, the Akaike's information criterion (AIC) and the root mean square error (RMSE). The model was optimized using the least square non-linear method and different versions were evaluated, differing in the number of parameters describing the reaction.

	All dependencies	No dependence on k_{off} and k_{bin}
a_{on}	$1.40 \times 10^{-5} \pm 8.40 \times 10^{-7}$	$9.96 \times 10^{-4} \pm 7.66 \times 10^{-5}$
b_{on}	$9.92 \times 10^{-3} \pm 6.00 \times 10^{-4}$	$5.45 \times 10^{-3} \pm 1.97 \times 10^{-4}$
c_{on}	$1.30 \times 10^{-1} \pm 6.87 \times 10^{-3}$	$1.68 \times 10^{-2} \pm 2.21 \times 10^{-3}$
a_{off}	$1.33 \times 10^{-5} \pm 4.10 \times 10^{-3}$	$4.57 \times 10^{-2} \pm 2.85 \times 10^{-3}$
b_{off}	$5.76 \times 10^{-3} \pm 1.52 \times 10^{-3}$	
c_{off}	$3.85 \times 10^{0} \pm 3.08 \times 10^{-2}$	
b_{clv}	$8.72 \times 10^{-1} \pm 4.00 \times 10^{-2}$	$2.61 \times 10^{-1} \pm 3.57 \times 10^{-3}$
c_{clv}	$3.34 \times 10^{-2} \pm 1.68 \times 10^{-3}$	$1.29 \times 10^{-1} \pm 2.74 \times 10^{-3}$
a_{rls}	$7.21 \times 10^{0} \pm 6.10 \times 10^{-1}$	$1.45 \times 10^{1} \pm 1.07 \times 10^{0}$
b_{rls}	$3.51 \times 10^{1} \pm 1.43 \times 10^{0}$	$1.85 \times 10^{2} \pm 1.64 \times 10^{1}$
c_{rls}	$3.83 \times 10^{-1} \pm 2.10 \times 10^{-2}$	$5.97 \times 10^{-1} \pm 4.98 \times 10^{-2}$
a_{bin}	$2.52 \times 10^{-1} \pm 8.98 \times 10^{-3}$	$9.75 \times 10^{-2} \pm 8.12 \times 10^{-3}$
b_{bin}	$9.36 \times 10^{-2} \pm 5.03 \times 10^{-3}$	$9.96 \times 10^{-4} \pm 7.66 \times 10^{-5}$
c_{bin}	$6.25 \times 10^{-1} \pm 4.19 \times 10^{-2}$	$5.45 \times 10^{-3} \pm 1.97 \times 10^{-4}$
**RMSE**	48.817	38.895
**AIC**	40721	38338

Table S8 Summary of the parameters describing the distribution of the exponential function parameters (a, b, and c) estimated for the different reaction steps. Equation 9 was used to obtain k_{rls} parameters, while Equation 10 was used for k_{on} and k_{clv}. The values were obtained from 100 randomly generated bootstrap data sets.

	Mean	Standard deviation	Lower bound (95 % CI)	Upper bound (95 % CI)	Skewness	Kurtosis
a_{on}	1.26×10^{-3}	2.20×10^{-5}	1.20×10^{-3}	1.29×10^{-3}	-2.68×10^{-1}	3.18×10^{-1}
b_{on}	2.33×10^{-3}	4.65×10^{-5}	2.23×10^{-3}	2.40×10^{-3}	-3.49×10^{-1}	-4.98×10^{-1}
c_{on}	2.98×10^{-2}	6.51×10^{-4}	2.84×10^{-2}	3.08×10^{-2}	-2.61×10^{-1}	-2.99×10^{-1}
k_{off}	5.48×10^{-2}	8.81×10^{-4}	5.32×10^{-2}	5.63×10^{-2}	-7.73×10^{-2}	-7.64×10^{-1}
b_{clv}	2.59×10^{-1}	4.10×10^{-3}	2.50×10^{-1}	2.65×10^{-1}	-6.98×10^{-1}	9.59×10^{-1}
c_{clv}	9.25×10^{-2}	9.63×10^{-4}	9.04×10^{-2}	9.42×10^{-2}	-4.90×10^{-1}	2.05×10^{0}
a_{rls}	1.64×10^{1}	2.62×10^{-1}	1.58×10^{1}	1.68×10^{1}	1.33×10^{-1}	-5.29×10^{-1}
b_{rls}	$2.39 \times 10^{+2}$	4.78×10^{0}	2.30×10^{2}	2.45×10^{2}	5.05×10^{-1}	1.02×10^{0}
c_{rls}	1.37×10^{-1}	1.69×10^{-3}	1.33×10^{-1}	1.39×10^{-1}	-3.39×10^{-2}	-5.99×10^{-1}
k_{bin}	1.00×10^{-1}	2.03×10^{-3}	9.73×10^{-2}	1.04×10^{-1}	6.89×10^{-1}	8.80×10^{-1}

	a_{on}	b_{on}	c_{on}	k_{off}	b_{clv}	c_{clv}	a_{rls}	b_{rls}	c_{rls}	k_{bin}
a_{on}	**1**	-0.4	-0.24	-0.33	-0.15	-0.08	0.28	0	0.16	-0.03
b_{on}	-0.4	**1**	-0.06	0.24	0.31	-0.16	-0.42	-0.41	-0.07	0.21
c_{on}	-0.24	-0.06	**1**	0.09	0.18	0.21	-0.13	-0.17	-0.05	-0.22
k_{off}	-0.33	0.24	0.09	**1**	0.01	-0.15	-0.38	-0.14	-0.07	-0.03
b_{clv}	-0.15	0.31	0.18	0.01	**1**	-0.18	-0.08	-0.07	-0.09	-0.24
c_{clv}	-0.08	-0.16	0.21	-0.15	-0.18	**1**	0.19	-0.01	-0.15	-0.08
a_{rls}	0.28	-0.42	-0.13	-0.38	-0.08	0.19	**1**	-0.15	-0.19	0.06
b_{rls}	0	-0.41	-0.17	-0.14	-0.07	-0.01	-0.15	**1**	0.24	-0.6
c_{rls}	0.16	-0.07	-0.05	-0.07	-0.09	-0.15	-0.19	0.24	**1**	-0.47
k_{bin}	-0.03	0.21	-0.22	-0.03	-0.24	-0.08	0.06	-0.6	-0.47	**1**

Figure S13 Correlation matrix of the exponential function parameters for the Mg^{2+} model.

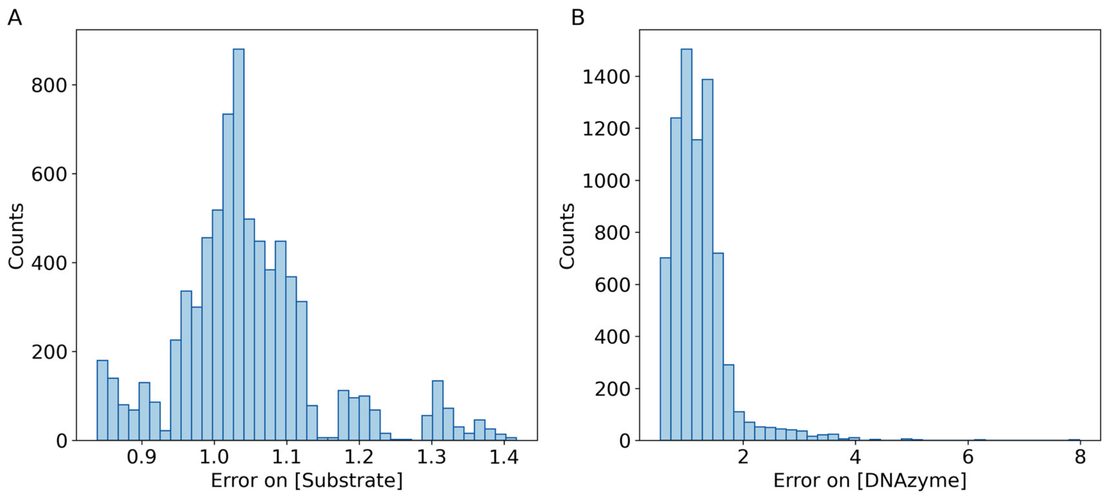

Figure S14 Histogram depicting the distribution of the relative error of the concentration of $Substrate_{Lg}$ **A**) and $DNAzyme_{Lg}$ **B**) obtained during the bootstrapping procedure. For each experimental curve in the data set, 100 simulated samples were obtained, and the errors were estimated for each of them. The histograms have been drawn based on 7500 examples.

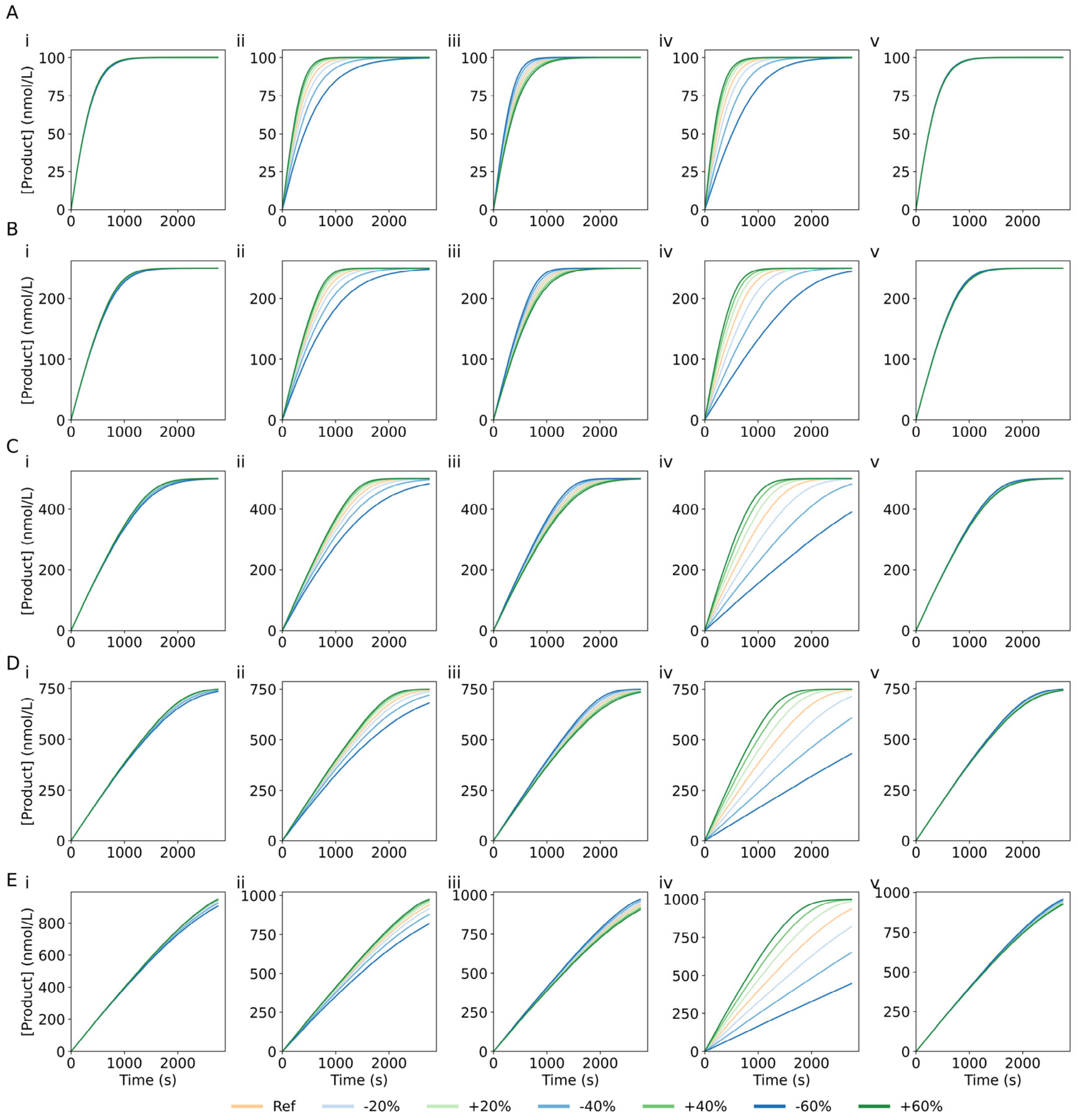

Figure S15 Simulation of the model output at 2 mmol/L of Mg^{2+} for different $Substrate_{Lg}$ concentrations: **A**) 100, **B**) 250, **C**) 500, **D**) 750 and **E**) 1000 nmol/L. To perform this analysis, the rate constants were systematically evaluated in a range of ± 60 % calculated from the estimate values shown in Table S8. The process was performed for each of the rate constant independently: (i) k_{on}, (ii) k_{off}, (iii) k_{clv}, (iv) k_{rls} and (v) k_{bin}.

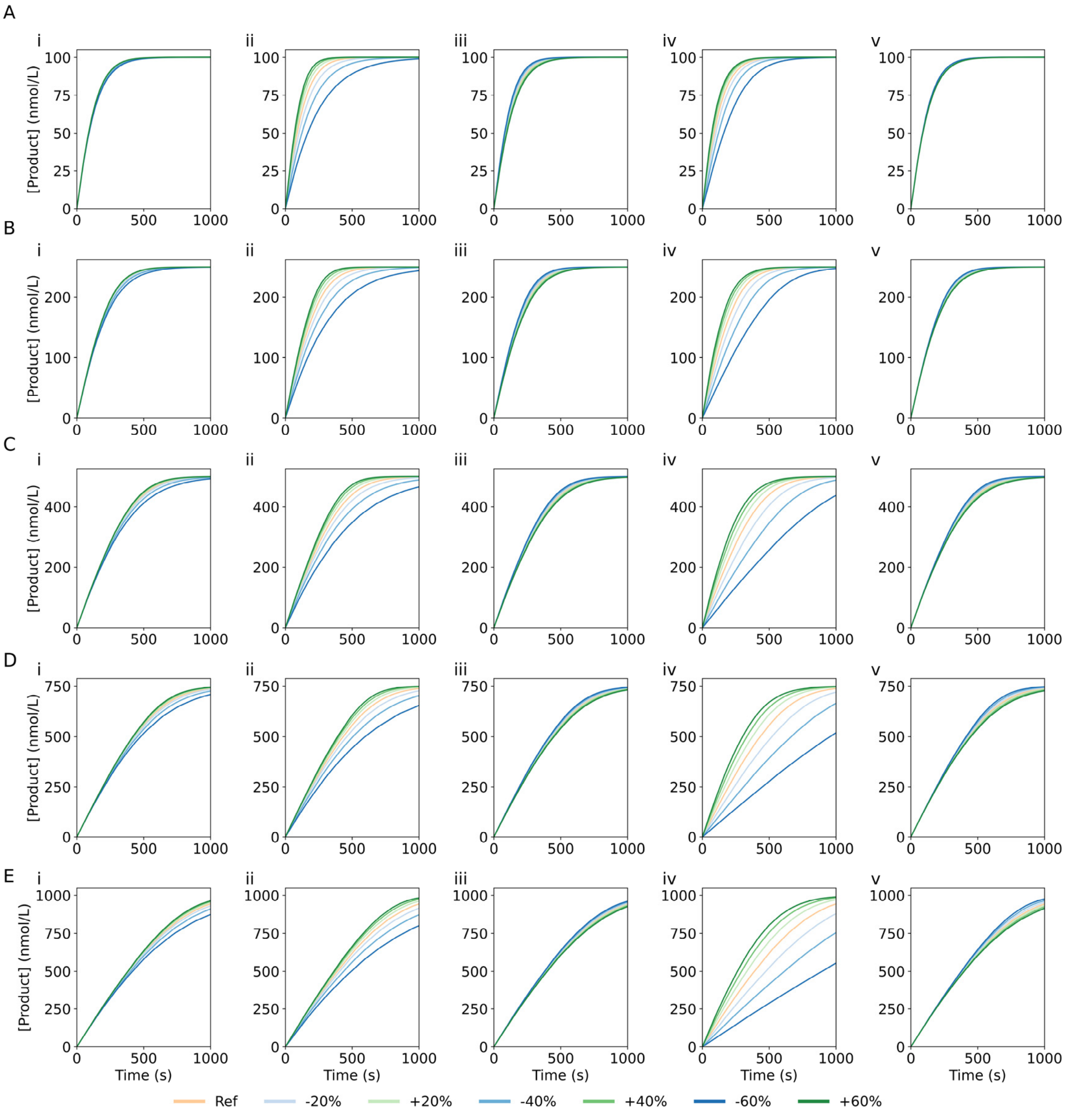

Figure S16 Simulation of the model output at 10 mmol/L of Mg^{2+} for different $Substrate_{Lg}$ concentrations: **A**) 100, **B**) 250, **C**) 500, **D**) 750 and **E**) 1000 nmol/L. To perform this analysis, the rate constants were systematically evaluated in a range of ± 60 % calculated from the estimate values shown in Table S8. The process was performed for each of the rate constant independently: (i) k_{on}, (ii) k_{off}, (iii) k_{clv}, (iv) k_{rls} and (v) k_{bin}. For visualization purposes the x-axis shows until 1000 s.

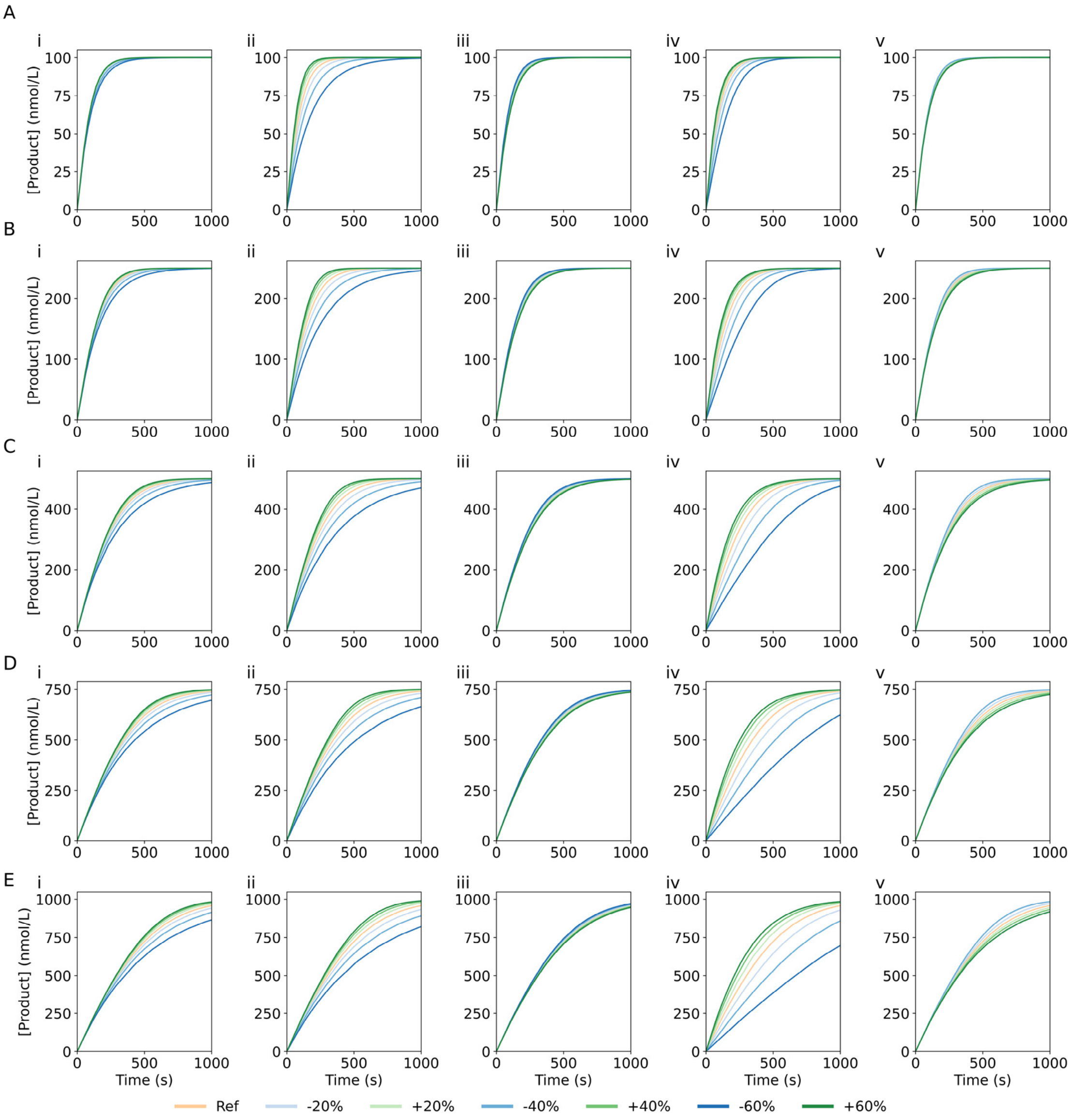

Figure S17 Simulation of the model output at 20 mmol/L of Mg^{2+} for different $Substrate_{Lg}$ concentrations: **A**) 100, **B**) 250, **C**) 500, **D**) 750 and **E**) 1000 nmol/L. To perform this analysis, the rate constants were systematically evaluated in a range of ± 60 % calculated from the estimate values shown in Table S8. The process was performed for each of the rate constant independently: (i) k_{on}, (ii) k_{off}, (iii) k_{clv}, (iv) k_{rls} and (v) k_{bin}. For visualization purposes the x-axis shows until 1000 s.

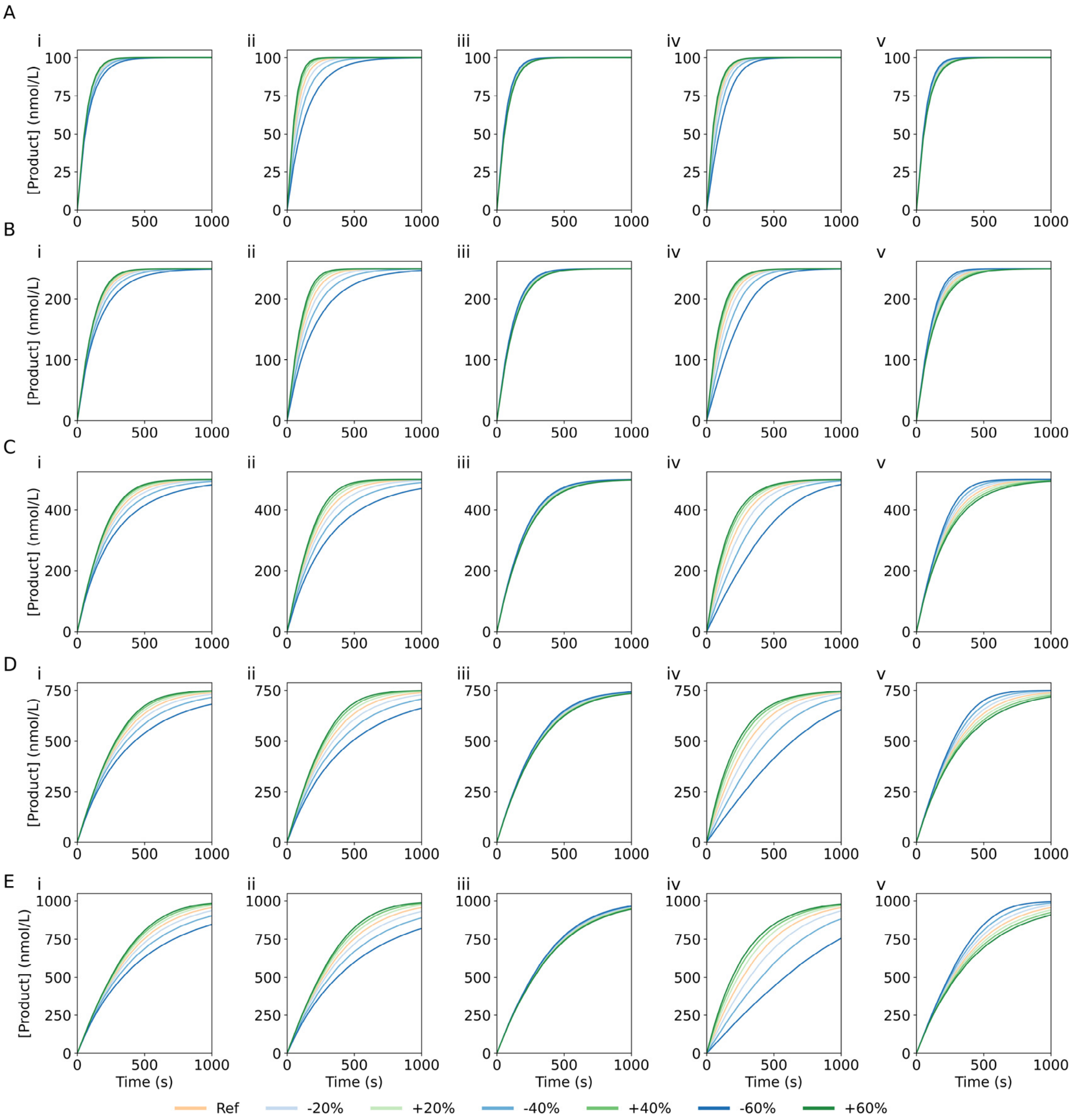

Figure S18 Simulation of the model output at 60 mmol/L of Mg^{2+} for different $Substrate_{Lg}$ concentrations: **A**) 100, **B**) 250, **C**) 500, **D**) 750 and **E**) 1000 nmol/L. To perform this analysis, the rate constants were systematically evaluated in a range of ± 60 % calculated from the estimate values shown in Table S8. The process was performed for each of the rate constant independently: (i) k_{on}, (ii) k_{off}, (iii) k_{clv}, (iv) k_{rls} and (v) k_{bin}. For visualization purposes the x-axis shows until 1000 s.

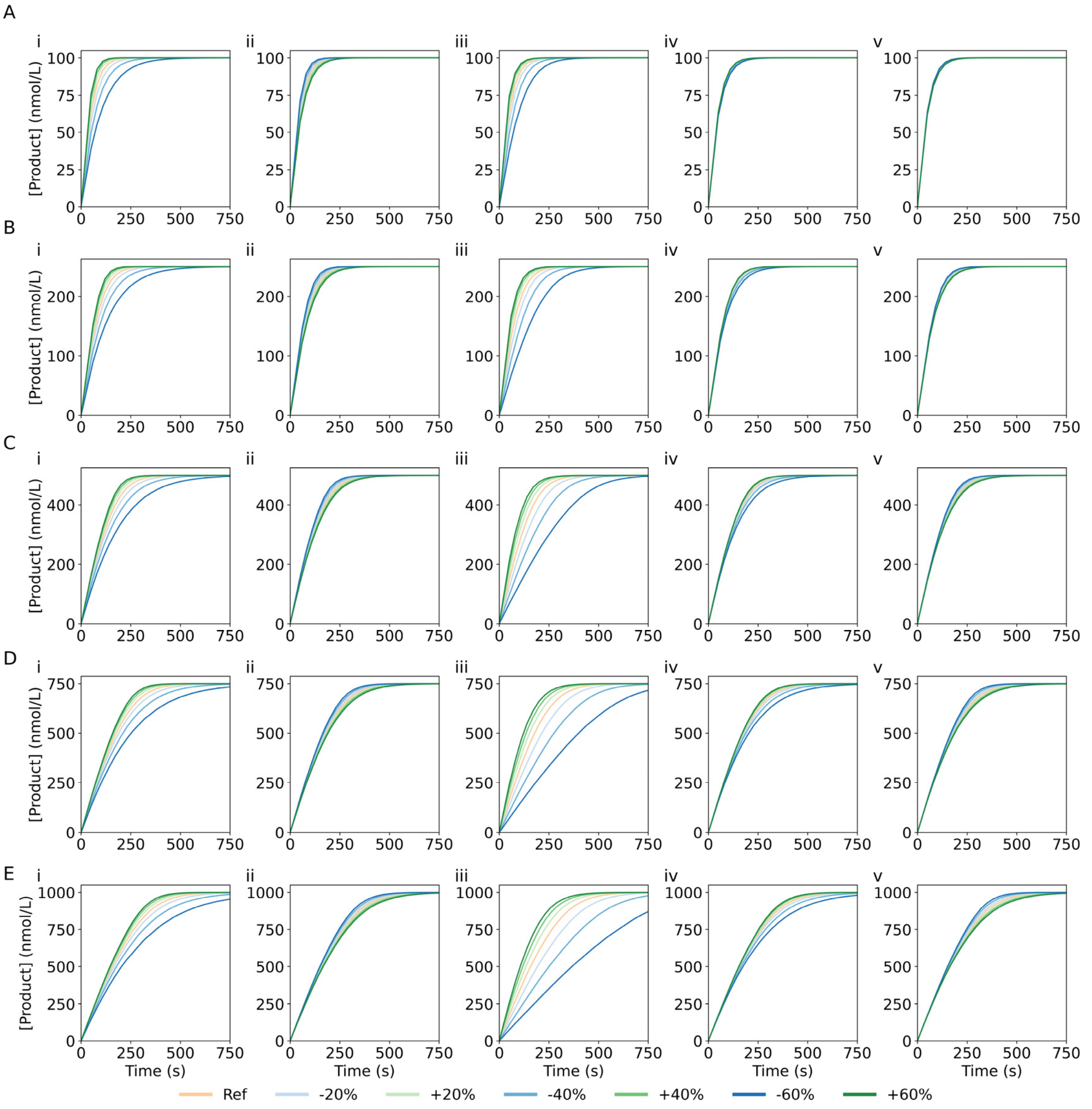

Figure S19 Simulation of the model output at 100 mmol/L of Mg^{2+} for different $Substrate_{Lg}$ concentrations: **A**) 100, **B**) 250, **C**) 500, **D**) 750 and **E**) 1000 nmol/L. To perform this analysis, the rate constants were systematically evaluated in a range of ± 60 % calculated from the estimate values shown in Table S8. The process was performed for each of the rate constant independently: (i) k_{on}, (ii) k_{off}, (iii) k_{clv}, (iv) k_{rls} and (v) k_{bin}. For visualization purposes the x-axis shows until 1000 s.

Table S9 Summary of the parameters describing the distribution of the rate constants obtained for the $DNAzyme_{Sh}$ from 100 randomly generated bootstrap data sets. For each experimental curve in the data set, 100 simulated samples were obtained, and the rate constants were estimated for each of them.

	Mean	Standard deviation	Lower bound (95 % CI)	Upper bound (95 % CI)	Skewness	Kurtosis
**k_{on} ((nmol/L)$^{-1}$ · s^{-1})**	4.00×10^{-3}	1.41×10^{-4}	3.73×10^{-3}	4.26×10^{-3}	3.69×10^{-2}	1.35×10^{-1}
**k_{off} (s^{-1})**	6.84×10^{-1}	2.33×10^{-2}	6.32×10^{-1}	7.33×10^{-1}	4.57×10^{-1}	1.27×10^{0}
**k_{clv} (s^{-1})**	2.62×10^{-1}	9.93×10^{-3}	2.38×10^{-1}	2.82×10^{-1}	-1.92×10^{-1}	9.58×10^{-1}
**krls (s^{-1})**	$1.55 \times 10^{+2}$	7.84×10^{0}	$1.38 \times 10^{+2}$	$1.70 \times 10^{+2}$	-2.97×10^{-1}	9.69×10^{-1}
**k_{bin} ((nmol/L)$^{-1}$ · s^{-1})**	5.96×10^{-2}	3.81×10^{-3}	5.16×10^{-2}	6.56×10^{-2}	-1.02×10^{-1}	-2.05×10^{-1}

	k_{on}	k_{on}	k_{clv}	k_{rls}	k_{bin}
k_{on}	**1**	0.48	-0.28	-0.52	0.1
k_{off}	0.48	**1**	-0.11	-0.07	-0.3
k_{clv}	-0.28	-0.11	**1**	0.47	0.02
k_{rls}	-0.52	-0.07	0.47	**1**	0.1
k_{bin}	0.1	-0.3	0.02	0.1	**1**

Figure S20 Correlation matrix of the rate constant estimates for DNAzyme$_{Sh}$ optimization.

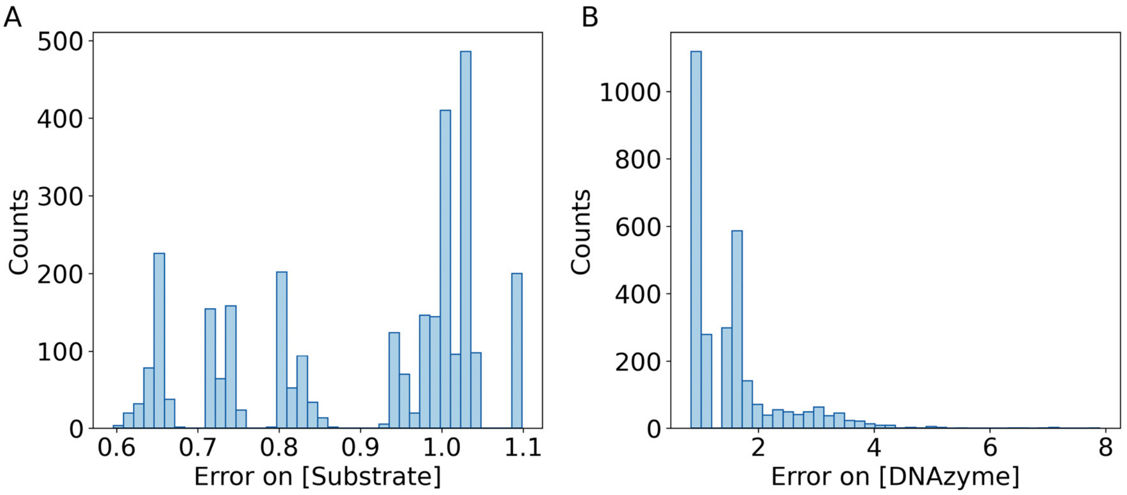

Figure S21 Histogram depicting the distribution of the relative error of the concentration of Substrate$_{Sh}$ **A**) and DNAzyme$_{Sh}$ **B**) obtained during the bootstrapping procedure. For each experimental curve in the data set, 100 simulated samples were obtained, and the errors were estimated for each of them. The histograms have been drawn based on 1500 examples.

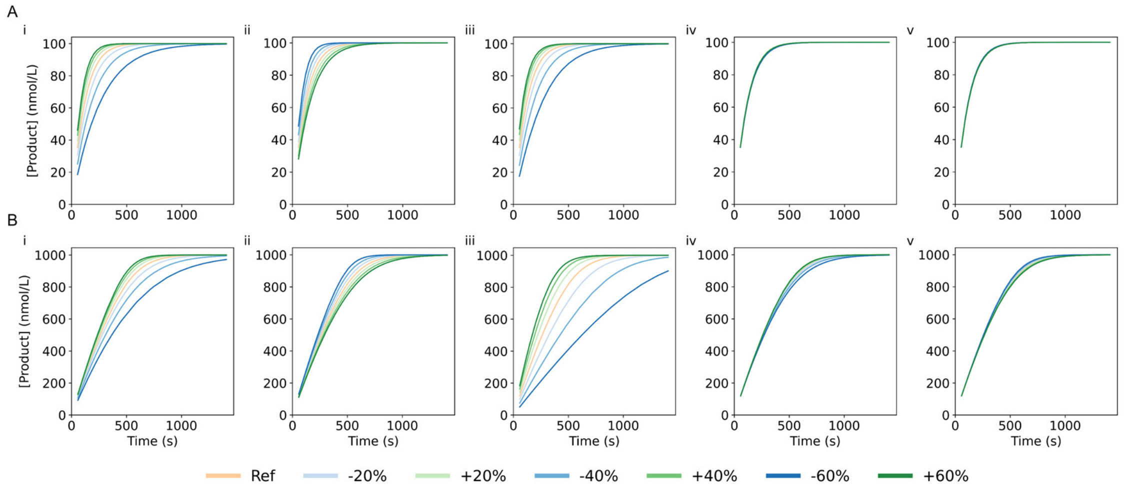

Figure S22 Simulation of the model output of DNAzyme$_{Sh}$ for **A**) 100 and **B**) 1000 nmol/L of Substrate$_{Sh}$. To perform this analysis, the rate constants were systematically evaluated in a range of ± 60 % calculated from the estimate values shown in Table S9. The process was performed for each of the rate constant independently: (i) k_{on}, (ii) k_{off}, (iii) k_{clv}, (iv) k_{rls} and (v) k_{bin}
